# Hierarchical nanostructure and synergy of multimolecular signalling complexes

**DOI:** 10.1038/ncomms12161

**Published:** 2016-07-11

**Authors:** Eilon Sherman, Valarie A. Barr, Robert K. Merrill, Carole K. Regan, Connie L. Sommers, Lawrence E. Samelson

**Affiliations:** 1 Racah Institute of Physics, The Hebrew University, Jerusalem 91904, Israel; 2 Laboratory of Cellular and Molecular Biology, CCR, NCI, NIH, Bethesda, Maryland 20892, USA

## Abstract

Signalling complexes are dynamic, multimolecular structures and sites for intracellular signal transduction. Although they play a crucial role in cellular activation, current research techniques fail to resolve their structure in intact cells. Here we present a multicolour, photoactivated localization microscopy approach for imaging multiple types of single molecules in fixed and live cells and statistical tools to determine the nanoscale organization, topology and synergy of molecular interactions in signalling complexes downstream of the T-cell antigen receptor. We observe that signalling complexes nucleated at the key adapter LAT show a hierarchical topology. The critical enzymes PLCγ1 and VAV1 localize to the centre of LAT-based complexes, and the adapter SLP-76 and actin molecules localize to the periphery. Conditional second-order statistics reveal a hierarchical network of synergic interactions between these molecules. Our results extend our understanding of the nanostructure of signalling complexes and are relevant to studying a wide range of multimolecular complexes.

## Abstract (web summary)

Imaging the nanostructure of signalling complexes in cells is challenging. Here, the authors present a multicolour, single-molecule, super-resolution approach with statistical tools to reveal a hierarchical topology and synergy of signalling complexes downstream of the T-cell receptor in fixed and live cells.

## Graphical abstract

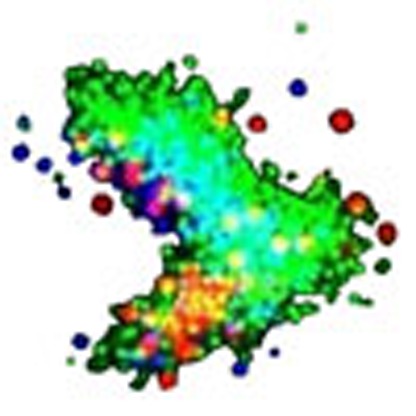

Signalling complexes are multimolecular structures containing adapter proteins and enzymes that form at the plasma membrane (PM) of cells following activation of multiple types of receptors^1^,^2^. They are the sites where signals controlling cellular processes are initiated and regulated. Signalling complexes are formed via stochastic, reversible molecular interactions that can give rise to a range of molecular stoichiometries and binding arrangements. Moreover, signalling complexes are often observed in larger aggregates and clusters that play a critical role in cell activation^1^,^3^,^4^,^5^. For instance, in the case of immune (T) cell activation, such multimolecular complexes and clusters trigger critical cell functions in response to the recognition of a cognate antigen by the T-cell receptor (TCR) with high levels of specificity and sensitivity^6^,^7^,^8^. However, in spite of their critical role in cell activation, the architecture and organization of signalling complexes and microclusters are poorly understood, as they are typically much smaller than the diffraction limit of light^9^,^10^. Recently, signalling complexes downstream of the TCR have been shown to comprise nanoscale structures whose sizes are best described as distribution functions, rather than deterministic unique structures^10^,^11^; that is, there is no unique crystal structure' to these complexes and their heterogeneity is best resolved at the single-molecule level. To better characterize the structure, topology and synergy in molecular interactions that govern the formation of signalling complexes, we developed an imaging technique that allows resolution of three different protein species within protein complexes at the single-molecule level. Our method relies on distinguishing photoactivatable (PA) fluorescent proteins (FPs) in fixed and living cells by differences in the energy needed for their photoactivation and by the separation in their spectral emission. Importantly, this approach minimized the potential misidentification of PAFPs seen in previous studies^12^,^13^, thus allowing us to study these molecules in close proximity within signalling complexes. We further developed statistical tools to resolve the mutual arrangement and synergy of protein interactions within signalling complexes nucleated by the critical adapter linker for activation of T cells (LAT)^14^. Using these novel imaging and statistical techniques, we demonstrate an intricate, hierarchical topology to LAT-nucleated clusters, and directly determine a network of synergic interactions at the single-molecule level within these clusters. The statistical methods we have employed can be used in the imaging studies of diverse molecular complexes found in various biological systems.

## Results

### Multicolour PALM imaging of signalling complexes

**Figure 1:**
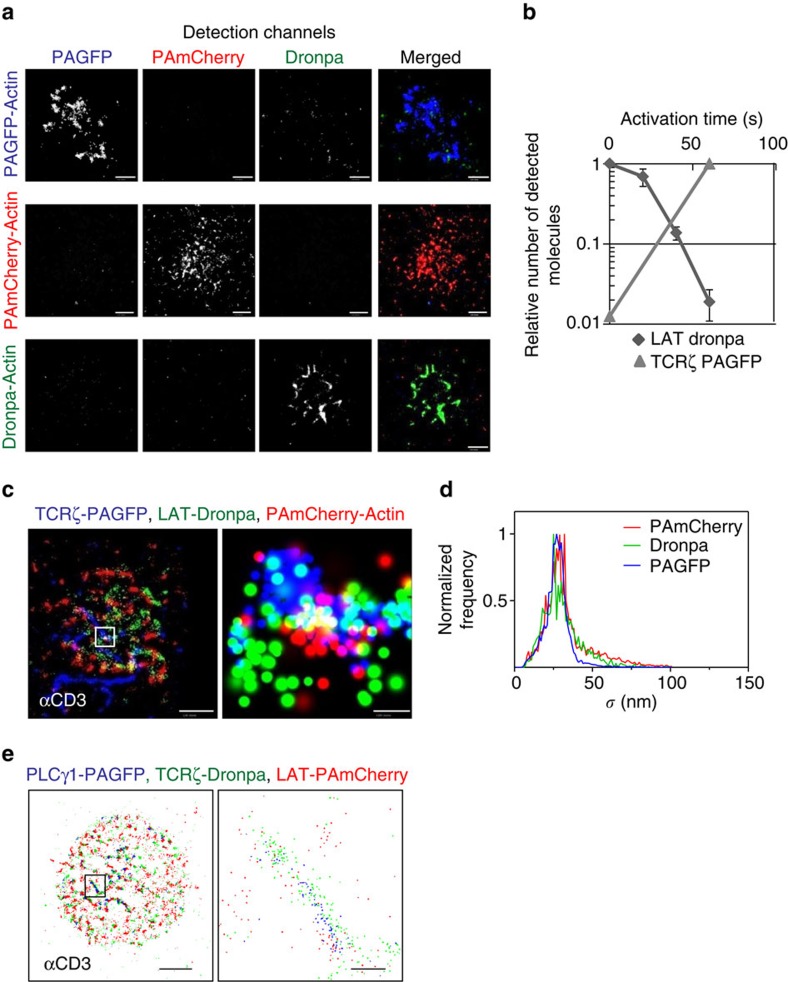
MC-PALM imaging of fixed and live T cells. (**a**) Multicolour PALM (MC-PALM) imaging of fixed E6.1 Jurkat cells expressing actin tagged with either PAGFP, Dronpa or PAmCherry, spread on an αCD3-coated coverslips for 3 min before fixation. For each construct, images are shown for the PAGFP (blue), PAmCherry (red) and Dronpa (green) channels separately and combined to show the extent of cross-talk between the channels. Maximal probability density values for PAGFP, Dronpa and PAmCherry rendering (see Supplementary Information for further details)—(**a**) 220, 50, 270 molecules per μm^2^ (top row); 140, 80, 90 molecules per μm^2^ (middle row); and 410, 450, 700 molecules per μm^2^ (bottom row), respectively. Scale bars, 2 μm. (**b**) The relative number of detected molecules of either LAT-Dronpa or TCRζ-PAGFP as a function of photoactivation dose, showing the cross-talk between the Dronpa and PAGFP channels in MC-PALM. (**c**) MC-PALM imaging of fixed Jurkat cells expressing TCRζ-PAGFP, LAT-Dronpa and PAmCherry-actin, spread on an αCD3-coated coverslips for 3 min before fixation. Maximal probability density values for PAGFP, Dronpa and PAmCherry rendering 260, 220, 220 molecules per μm^2^. Scale bar, 2 μm (left); and 200 nm (right). (**d**) The distribution of error (1σ) in localization of individual PAGFP, PAmCherry and Dronpa molecules imaged via MC- PALM. (**e**) MC-PALM of a live Jurkat E6.1 cell expressing PLCγ1-PAGFP, TCRζ-Dronpa and LAT-PAmCherry on an αCD3-coated coverslip. Scale bar, 2 μm (left); and 200 nm (right).

Multicolour photoactivated localization microscopy (MC-PALM) imaging holds the promise of resolving the structure and composition of signalling complexes in molecular detail in intact cells and at nanometre resolution. However, at present, the spectral emissions of PA proteins that are used in PALM typically fall into either a green (GFP-like, 515–530 nm) or a red (RFP-like, 550–600 nm) channel (for example, refs 15, 16). Identification of PA proteins with overlapping emission spectra for multicolour PALM imaging has been demonstrated^12^,^13^. However, these studies resulted in significant cross-talk, that is, misidentification of the PA proteins, which is detrimental to the study of the intricate structure of signalling complexes. We have found that PAGFP and Dronpa^17^ strongly differ, by a factor of ∼50, in the intensity of 405 nm light required for their photoactivation using a confocal microscope (Fig. 1b). In addition, Dronpa can be activated by 340 nm light, which fails to activate PAGFP. Using this difference in activation energy, we developed an imaging approach to sequentially image Dronpa- and PAGFP- tagged proteins using a wide-field total internal reflection (TIRF) microscope capable of PALM imaging (Fig. 1a; see details in Methods section). We first imaged Dronpa using low-intensity photoactivation light and then stepwise increased the PA energy for selective imaging of PAGFP. The increased photoactivation light also activated PAmCherry. PAGFP and PAmCherry could then be separated by their distinct emission spectra (Supplementary Table 1). Cross-talk between the channels was <2% for all channels (Fig. 1a,b). We refer to this method as MC-PALM and demonstrated its utility in imaging signalling complexes by visualizing a TCR subunit (TCRζ-PAGFP), LAT-Dronpa and PAmCherry-actin in fixed E6.1 Jurkat cells (Fig. 1c). The error in localization accuracy (1σ) of these molecules peaked at ∼25 nm for all three molecules (Fig. 1d). We further demonstrated the efficacy of MC-PALM for live-cell imaging in E6.1 Jurkat cells that expressed PLCγ1-PAGFP, TCRζ-Dronpa and LAT-PAmCherry (Fig. 1e and Supplementary Table 1).

### Synergic recruitment of effectors to LAT clusters

**Figure 2:**
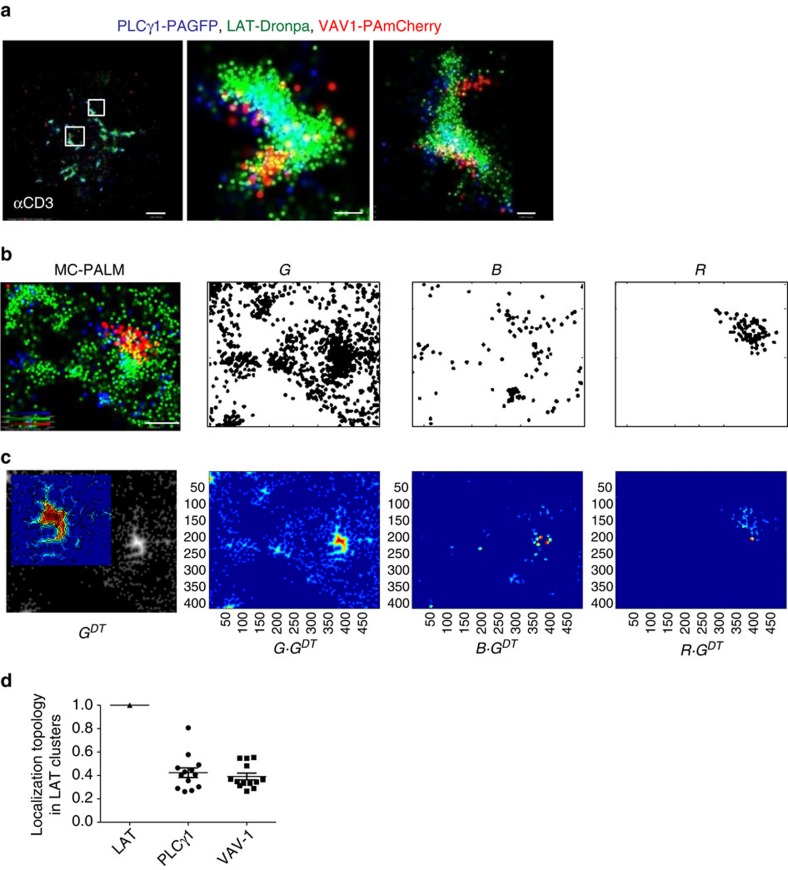
An intricate structure in the recruitment of effector proteins to LAT clusters. (**a**) MC-PALM imaging of fixed Jurkat cells expressing PLCγ1-PAGFP (blue), LAT-Dronpa (green) and VAV1-PAmCherry (red), spread on an αCD3-coated coverslips for 3 min before fixation. Two zoomed images of representative LAT clusters are shown. Maximal probability density values for PAGFP, Dronpa and PAmCherry rendering 370, 740, 280 molecules per μm^2^, respectively. Scale bar, 2 μm (left); and 200 nm (middle and right). (**b**) Sequence used to calculate localization topology (see Analyses section in Supplementary Information for further details): an MC-PALM image (left) is separated into three channels (that is, right images, labelled *G,B,R*), where single molecules are represented as 20 nm black disks. Maximal probability density values for PAGFP, Dronpa and PAmCherry rendering 270, 460, 140 molecules per μm^2^, respectively (colour bars in the left panel represent probability densities). Scale bar, 0.5 μm. Note that these imprints are shown as aids to the reader and did not affect any of the analyses. (**c**) A distance transform is performed for the green LAT channel, generating an image *G*^*DT*^ (left), where the intensity increases with distance from the cluster boundary. An inset in the *G*^*DT*^ image shows a magnified view of a LAT cluster in *G*^*DT*^ using heat map colour coding. The binary images from all three channels are multiplied by *G*^*DT*^ to produce weighted images of the binary images *G*, *B* and *R* (right images; images rendered using heat map colour coding). (**d**) Relative localization topologies (the average intensity of a weighted binary image divided by the average intensity of the LAT-weighted binary image) for the three channels of multiple cells (*n*=13) expressing LAT-Dronpa, PLCγ1-PAGFP and VAV1-PAmCherry rendered using heat map colour coding (see Supplementary Information for further details).

We next employed MC-PALM to study multimolecular signalling complexes that are critical for T-cell activation and function. Following TCR stimulation, multiple effector proteins bind LAT at phosphorylated tyrosine residues, thus triggering critical signalling pathways in a matter of seconds^14^. The effector enzyme PLCγ1 and the guanine nucleotide exchange factor VAV1, two important proteins that are recruited to LAT, closely interact^18^, and play critical roles in the control of Ca^++^ influx, actin polymerization and transcription of the nuclear factor of activated T cells^19^. We set out to resolve how these proteins are recruited to LAT using MC-PALM, and then quantified their synergy in interactions and positioning within LAT-based clusters, as explained below. We first imaged fixed E6.1 cells stably expressing PLCγ1-PAGFP and transiently expressing LAT-Dronpa and VAV1-PAmCherry (Fig. 2a). These cells were stimulated for 3 min on coverslips coated with antibodies that engage the TCR (αCD3), resulting in T-cell activation and cell spreading^20^. In the MC-PALM images, VAV1 and PLCγ1 showed recruitment to LAT clusters in closely positioned domains (Fig. 2a). This result demonstrates our ability to directly capture an intricate nanoscale organization of signalling molecules within complexes at the single-molecule level. This pattern could not be captured by other techniques and could only be inferred from previous studies^14^,^18^.

These images, showing well-resolved positions of individual molecules of multiple species, must be properly analysed to understand the structure and topology of signalling complexes. It has been a challenge to find statistical methods that can quantify the relationships in the resultant three-colour point patterns. Univariate and bivariate second-order statistics have been used to describe the molecular organization at the PM of cells^21^,^22^. Specifically, the second-order statistics of bivariate pair correlation function (PCF) or Ripley's K-functions^23^ quantify in a point pattern how the density of points (or molecules) of one species varies as a function of distance from all points (or molecules) of a second species. However, such previously used second-order statistics do not contain information about the topology of clusters. Colocalization measures (Pearson's or Mander's^24^) also cannot capture topological information and are not suited for point patterns.

Hence, we developed a method that can quantify the topology of LAT clusters (Fig. 2b,c), as detailed below and in the Analyses section of Supplementary Information. Starting from the three-colour rendering (Fig. 2b, left image), we first generated three binary images matching the three channels imaged by MC-PALM (*G*, *B* and *R* in Fig. 2b). In each binary image, a disk with a diameter equivalent to 20 nm marked the positions of individual molecules (see consideration for choosing the disk size in the Analyses section of Supplementary Information). The intensity inside a disk was set to 1, while areas outside disks without molecules were assigned a value of 0. In areas where disks overlapped, the intensity remained 1. We then used a distance transform^25^ of the binary image (or actually, its negative) to form a topographic map of LAT complexes from the LAT channel (Fig. 2c, lower left panel and its inset; see definitions in equations (1) and (2) and further details in the Analyses section of Supplementary Information). The distance transform labels each pixel of the image with the distance to the nearest boundary pixel in a binary image (here, the boundary is the edge of the cluster that contains the pixel). In the resultant watershed or topographical image (*G*^*DT*^) intensities are highest at points furthest from the boundaries in the binary image. Finally, weighted images were produced by multiplying each binary image (namely *G*, *B* and *R*) by the watershed image *G*^*DT*^ (Fig. 2c, three right images labelled *G·G*^*DT*^, *B·G*^*DT*^ and *R·G*^*DT*^).

We also developed a measure we call the ‘localization topology' to quantify the extent to which other proteins are recruited to the centres of LAT clusters (Fig. 2d). The localization topology is computed by dividing the average intensity from each weighted binary image (Fig. 2c, three right images) by the average intensity from the LAT-weighted image (Fig. 2c, image labelled *G·G*^*DT*^). Thus the localization topology of LAT is set to 1 and typical values for other molecules range from 0 to 1. Molecules that tend to be recruited to the centres of LAT clusters have higher values than those recruited to the periphery of clusters. Note that localization topology of over 1 is possible in cases where a specific molecular species is enriched at the centre of clusters, with respect to the molecular species that defines the cluster; however, we did not observe such a case in our experiments. Similar localization topologies were found for PLCγ1 and VAV1 in individual and multiple cells (Fig. 2d), indicating that the two molecules were found in similar distances from the centres of LAT clusters (that is, in similarly intense regions of the LAT watershed).

**Figure 3:**
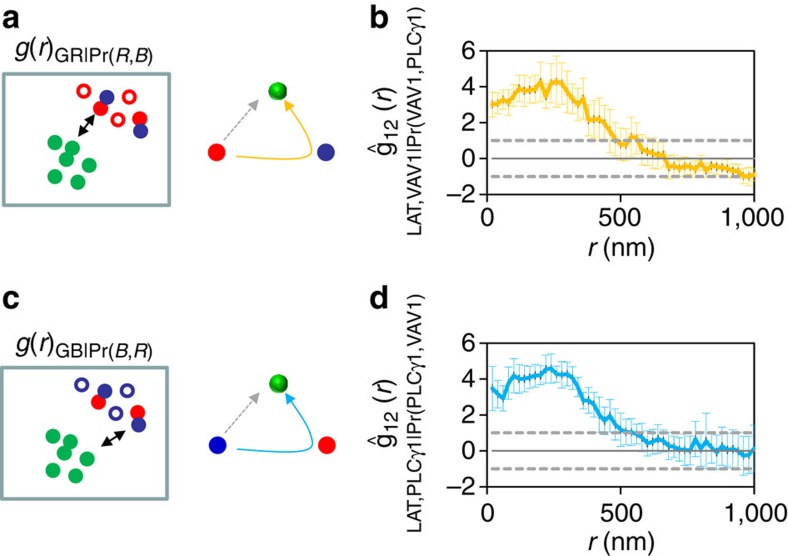
Mutually synergic recruitment of effector proteins to LAT clusters. (**a**, left) A scheme depicting the analyses of interaction synergy between molecules of three types. Red molecules are selected based on their proximity to blue molecules (selected molecules are marked with filled circles while unselected molecules are marked with empty circles). (right) A scheme of molecular interactions, summarizing the hierarchical pattern of molecular interactions under study by the statistics on left. Yellow arrow indicates synergic binding, while dotted grey arrow mark direct binding. (**b**) A conditional bivariate PCF analysing the synergic interaction of VAV1 and PLCγ1 upon the binding of VAV1 to LAT. A conditional bivariate PCF was first generated for the selected set with respect to green molecules (see definition in equation (6) in Supplementary Information). The PCF was compared with PCFs due to the 19 random sets generated by Monte-Carlo simulations. The conditional biavariate PCFs were standardized and averaged for multiple cells (*n*=13, same samples used in **c**, we further refer to this curve as a standardized conditional bivariate PCF. See equations (7–10) in Supplementary Information for definition). The flat dotted lines mark the 95% confidence interval due to the Monte-Carlo simulations. The portion of the PCF (in yellow) above the dotted lines indicates significant interaction synergy at lower *r* values. (**c**, left) A scheme depicting the analyses of interaction synergy between molecules of three types, namely the recruitment of blue molecules to green molecules through binding to red molecules (filled blue circles mark selected molecules). (right) A scheme of molecular interactions, summarizing the hierarchical pattern of molecular interactions under study in the statistics on left. Cyan arrow indicates synergic binding while dotted grey arrow mark direct binding. (**d**) A conditional bivariate PCF analysing the interaction synergy of PLCγ1 and VAV1 upon the binding of PLCγ1 to LAT. The portion of the curve (in cyan) above the dotted lines indicates significant interaction synergy. Error bars are s.e.m.

Cooperative binding, where multiple binding events enhance the affinities of interaction, is a hallmark of multimolecular interactions found in enzymatic reactions^26^, receptor triggering^27^,^28^,^29^ and in signal transduction via heterogeneous and dynamic molecular complexes. Our ability to image three molecular species at once allowed us to characterize complex multimolecular interactions and examine binding synergy (interpreted here as cooperativity) or competition. To overcome the limitations of standard second-order statistics to resolve these issues, we expanded these methods to analyse trivariate interactions, employing what we term conditional second-order statistics (Fig. 3; see section on Analyses in Supplementary Methods for further details). These statistics allow us to quantify any increase or decrease in the propensity of two molecules to be adjacent to one another in the presence of a third molecule. We use the term synergic interaction to refer to all cases where proximity to one type of molecule increases the probability that our molecule of interest will also be close to a second type of molecule. It does not imply that the species are binding together or increasing actual binding affinities. Such an interpretation is limited to cases where direct physical binding was verified by biochemistry or FRET, which is the case for all molecules involved in this study^18^,^30^,^31^. Since we are specifically interested here in the formation of LAT clusters, this kind of synergy allows us to document when a molecule is recruited to a cluster whether it is binding directly to LAT or indirectly via interaction with another molecule that binds to LAT (see a more general description of the interaction synergy analyses in the Analyses section of Supplementary Methods). We start by selecting a subpopulation of the molecules of one species, based on their proximity to a second species. In this example shown in cartoon form in Fig. 3a, we selected VAV1 molecules (shown in red) that were close to PLCγ1 molecules (shown in blue). Close proximity was defined with a distance threshold of 40 nm after testing a range of thresholds (see also Supplementary Fig. 1 and as explained in Supplementary Methods). We then employed bivariate PCF to describe the interaction of that selected subpopulation of the first species with a third molecular species (see equation (6) in Supplementary Methods). In this example, LAT was the third species (shown in green in the cartoon). The relevant null hypothesis was that the proximity-based selection of the molecules of the first species was due to purely random sampling. In such a case, the resultant PCF of the selected set of the first species interacting with the third species should not be different than the PCF of any other randomly chosen, equally large set. That is, if the proximity of VAV1 to PLCγ1 had no effect on the interaction between the selected VAV1 molecules and LAT, then the PCF of the proximity-based set of VAV1 molecules and LAT should be the same as the PCF of any randomly chosen set of an equal number of VAV1 molecules and LAT. The right panel of Fig. 3a shows a summary of the hierarchical pattern of molecular interactions under study. The dotted grey arrow indicates a direct interaction between VAV1 and LAT, while the curved arrow shown in yellow indicates a synergic interaction where VAV1 molecules in close proximity to PLCγ1 also tend to be in close proximity to LAT molecules. Similar cartoons are used in the rest of the work to compactly describe various patterns and scenarios of molecular interaction. We used Monte-Carlo simulations to derive 19 such sets of randomly chosen VAV1 molecules and generated their bivariate PCFs with LAT. The values of these simulations defined the 95% confidence interval of the random process. The conditional bivariate PCFs of the experimental data and simulations were standardized (see equations (7)–(10) in Supplementary Methods for definition) and averaged for multiple cells (*n*=13, Fig. 3b). After this step, the flat dotted lines mark the 95% confidence interval of random interactions modelled by the Monte-Carlo simulations. A PCF lying above the confidence intervals indicates that the interaction of the selected molecules of species 1 to species 3 is promoted by the proximity of species 1 and 2. Conversely a value below the range would indicate negative synergy of the interaction. Using this analysis we found that positive synergy exists in the binding of VAV1 to LAT clusters via their mutual interaction with PLCγ1 (Fig. 3b) and in the binding of PLCγ1 to LAT clusters via PLCγ1 interactions with VAV1 (Fig. 3c,d). The interaction synergy in both cases was significant up to ∼500 nm. Taken together, we conclude from these results that VAV1 and PLCγ1 are recruited to LAT clusters in a mutual and synergic manner that gives rise to closely related nanoscale molecular patterns with respect to LAT. Thus, conditional bivariate second-order statistics (PCF) can be utilized to describe trimolecular interactions and synergies (interpreted in this specific case as cooperativity) within multimolecular structures.

### Hierarchical recruitment of PLCγ1 and SLP-76 to LAT clusters

The question arises whether the synergy of molecular interactions in signalling complexes is always symmetrical and mutual. To address this, we studied a second set of molecules within LAT-nucleated complexes, namely PLCγ1, LAT and SLP-76. SLP-76 is a critical adapter recruited to LAT complexes through its constitutive interaction with the SH2 and SH3 domain-containing adapter protein Gads. Gads interacts with SLP-76 through an SH3 domain and also binds to phosphorylated LAT. Previous studies have indicated that interactions between SLP-76 and PLCγ1 stabilize their binding to LAT complexes^18^. SLP-76 also links LAT complexes to the actin cytoskeleton via interactions with Nck, VAV1 and Itk^30^. We showed recently that SLP-76 preferentially localizes to the periphery of LAT clusters^10^, although this unexpected arrangement cannot be explained by previous results^14^,^30^,^32^.

**Figure 4:**
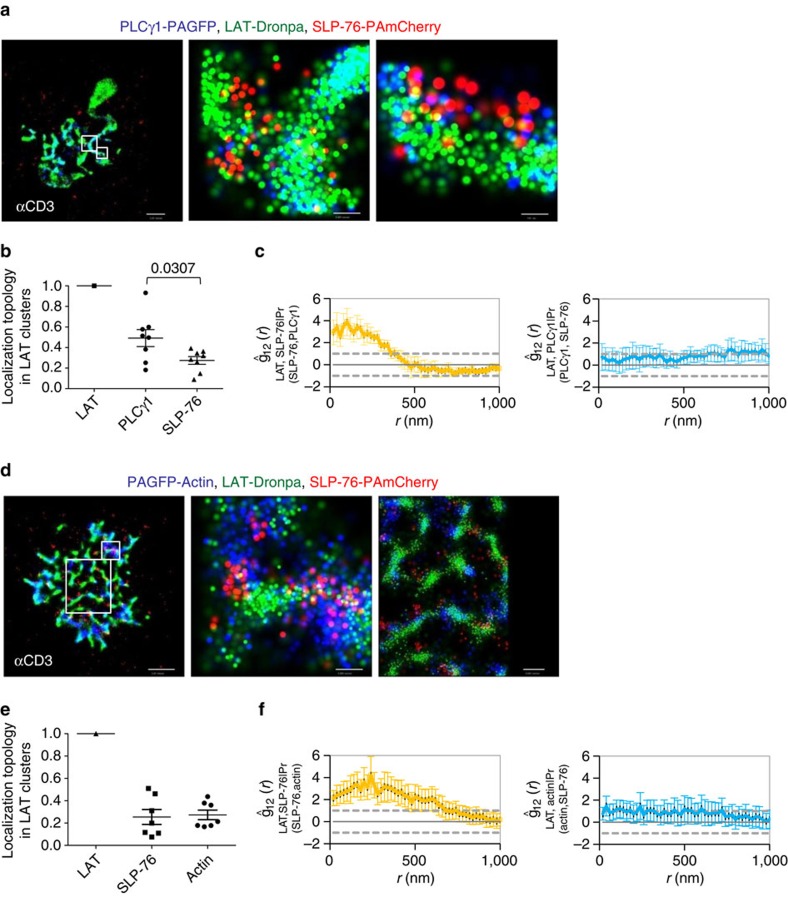
Hierarchical recruitment of PLCγ1, SLP-76 and actin to LAT clusters. (**a**–**c**) Hierarchical recruitment of PLCγ1 and SLP-76 to LAT clusters. (**a**) MC-PALM imaging of fixed Jurkat cells expressing PLCγ1-PAGFP (blue), LAT-Dronpa (green) and SLP-76-PAmCherry (red), spread on an αCD3-coated coverslips for 3 min before fixation. Two zoomed images of representative LAT clusters are shown. (**b**) Topology analyses in multiple cells (*n*=8) imaged as in **a**. *P* value was calculated using a two-tailed *t*-test for unequal variances. (**c**) A conditional bivariate PCF (standardized) analysing the interaction synergy of SLP-76 and PLCγ1 on the binding of SLP-76 to LAT (left) or the interaction synergy of PLCγ1 and SLP-76 on the binding of PLCγ1 to LAT (right). Maximal probability density values for PAGFP, Dronpa and PAmCherry rendering—(**a**) 190, 390, 340 molecules per μm^2^, respectively. Scale bars, 2 μm (left); 200 nm (middle); and 100 nm (right). (**d**–**f**) Actin promotes the nanoscale patterning of LAT clusters. (**d**) MC-PALM imaging of fixed Jurkat cells expressing PAGFP-actin (blue), LAT-Dronpa (green) and SLP-76-PAmCherry (red), spread on an αCD3-coated coverslips for 3 min before fixation. Two zoomed images of representative LAT clusters are shown. Maximal probability density values for PAGFP, Dronpa and PAmCherry rendering 310, 330, 290 molecules per μm^2^, respectively. Scale bar, 2 μm (left); 200 nm (middle); and 500 nm (right). (**e**) Topology analyses of molecular patterning in multiple cells imaged as in **a** (*n*=7). (**f**) A conditional bivariate PCF (standardized) analyses of the interaction synergy of SLP-76 and actin on the binding of SLP-76 to LAT (left) or the interaction synergy of actin and SLP-76 on the placement of actin with respect to LAT (right). Error bars are s.e.m.

Using MC-PALM, we imaged the interactions between PLCγ1-PAGFP, LAT-Dronpa and SLP-76-PAmCherry in fixed E6.1 cells spread on TCR-activating coverslips for 3 min. Our imaging recaptured the efficient recruitment of PLCγ1 to LAT clusters and the recruitment of SLP-76 to the periphery of clusters containing LAT (Fig. 4a)^10^. Topology measurements of both PLCγ1 and SLP-76 with respect to LAT showed significant quantitative differences. PLCγ1 was significantly closer to LAT than SLP-76 (Fig. 4b). By our standardized conditional PCF analyses, we found that a subset of SLP-76 molecules, proximal to PLCγ1, showed a significantly increased interaction with LAT in comparison with the null hypothesis of random selection (Fig. 4c, left). Surprisingly, we found that the selected subset of PLCγ1 molecules proximal to SLP-76 were not different in their interaction with LAT than any other randomly selected subset (Fig. 4c, right). Reversing the fluorescent tags on PLCγ1 and SLP-76 generated similar results (Supplementary Fig. 2). These results indicate that although SLP-76 recruitment to LAT could be affected by PLCγ1, the recruitment of PLCγ1 to LAT is independent of SLP-76.

### Actin promotes the recruitment of SLP-76 to LAT

Actin has been proposed to play a crucial role in patterning the immune synapse (IS)^33^. LAT signalling complexes are linked to actin polymerization by proteins that bind to SLP-76 (ref. 30). Recently, we showed that the disruption of the link between SLP-76 and polymerized actin abrogated SLP-76 recruitment to the periphery of LAT clusters and affected SLP-76 translocation^10^. To further study the relationship of LAT clusters to actin via SLP-76, we used MC-PALM to image fixed E6.1 Jurkat cells expressing PAGFP-actin, LAT-Dronpa and SLP-76-PAmCherry, spread on TCR-activating coverslips for 3 min. Our images show that actin interdigitated with LAT clusters but was more co-localized with SLP-76 at the periphery of LAT clusters, both on TCR-activating coverslips (Fig. 4d) and on TCR-non-activating coverslips, where SLP-76 was poorly recruited^7^,^10^ (Supplementary Fig. 3). The measures of localization topology of the two molecules were similar (Fig. 4e and Supplementary Fig. 3B), and significantly lower than those of PLCγ1 (Fig. 4e; *P*=0.0174) and VAV1 (Fig. 4e; *P*=0.0226). The *P* values were calculated using a two-tailed *t*-test for unequal variances. These results demonstrate that actin surrounds LAT clusters with a topology similar to the topology of SLP-76 around LAT. By our conditional PCF analyses, we found a subset of SLP-76 molecules, proximal to actin that showed an increased interaction with LAT, beyond the random selection null hypothesis (Fig. 4f, left). Surprisingly, however, the actin molecules selected for their proximity to SLP-76, did not show a difference in their interaction with LAT compared with the random selection hypothesis (Fig. 4f, right). These results indicate that SLP-76 binding to LAT could be promoted by the presence of actin, but that the localization of actin around LAT was not affected by the presence of SLP-76.

### Robustness of the interaction synergy and topology statistics

We tested the robustness of our statistical tools for two potential issues. First, we examined the ability of our statistics to correctly distinguish the case of positive molecular interaction synergy from other cases, in which no synergic effect was present (negative cases). Second, we studied the robustness of our statistics to potential experimental artefacts, including cross-talk and misidentification of imaged molecular species, partial undercounting (undersampling) or over-counting (over-sampling), aggregation of molecules that participate in recruitment and localization errors. Since it is complicated to address the multitude of potential scenarios and artefacts experimentally, we turned to statistical simulations to account for these issues, as detailed below.

**Figure 5:**
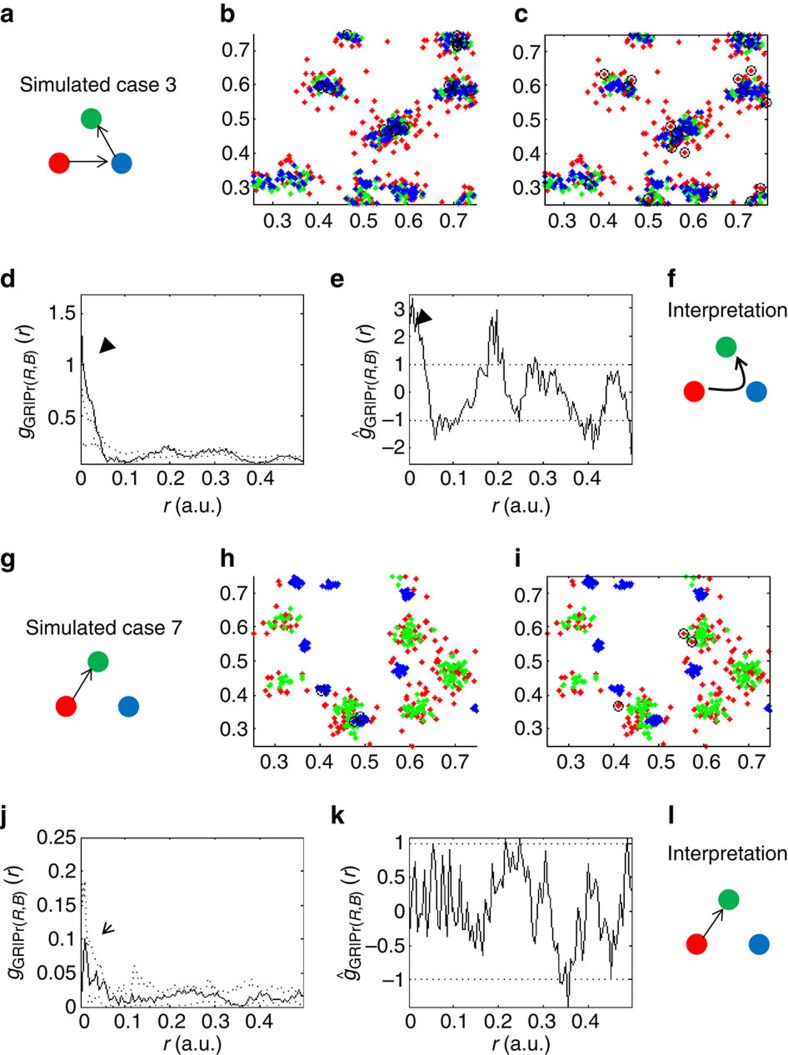
The synergy analysis distinguishes synergic and non-synergic molecular interactions. (**a**–**f**) A case of positive interaction synergy: simulated data and interaction synergy analyses of a case depicted in **a** (case 3 in Supplementary Table 2). (**a**) The model of molecular interactions where red molecules (red filled circles) were recruited to blue molecules (blue filled circles) that are themselves recruited to a reference species of green molecules (green filled circles). Thus, blue molecules were ‘directly' recruited to green molecules, while red molecules were ‘indirectly' recruited to green molecules. (**b**) A representative simulated data (*n*=20). (**c**) A representative realization of molecular clustering due to one of 19 Monte-Carlo simulations (conducted for each simulated data in **b**), where red molecules were selected randomly. (**d**) The conditional bivariate PCF of the simulated data in **b** (see definition in equation (6) in Supplementary Information), where dotted lines mark the 95% confidence interval due to the Monte-Carlo sets (as shown in **c**). Significant interaction synergy is highlighted with a black arrowhead. (**e**) The standardized conditional PCF (that is, the standardized form of the curve in **d** as defined in equations 7–10 in Supplementary Information). Significant interaction synergy is highlighted with a black arrowhead. (**f**) The resultant molecular interaction scheme based on the interpretation of the conditional PCFs in **d**,**e**. Synergic recruitment of red molecules to green molecules through blue molecules is marked with a curved arrow between the interacting species. (**g**–**l**) An example of a case of no interaction synergy: Simulated data and interaction synergy analyses of a case depicted in **g** (case 7 in Supplementary Table 2). (**g**) The model of molecular interactions, where red molecules are directly recruited to green molecules (red and green filled circle), while the positioning of blue molecules (blue filled circles) was unrelated to the red and green species (compare with scheme of **a**. (**h**–**i**) As for **a**–**f** of this figure. The straight arrow in **l** marks the direct recruitment of red molecules to green molecules, with no influence by blue molecules.

Our experimental measurements in this study were focused on the recruitment of molecules to LAT clusters. Naturally, our experiments could not explore all of the possible molecular interaction scenarios that are needed for the complete experimental validation of our statistics of interaction synergy and localization topology. Hence, we turned to simulations and studied all possible scenarios of trimolecular interactions that relate to a specific reference molecule (see Supplementary Table 2, Fig. 5 and Supplementary Figs 4–6). Specifically, we examined whether our proposed statistics were able to capture synergic interactions and reject non-synergic interactions for all simulated scenarios. The molecular patterns of interactions are depicted in the interaction schemes in panels a and g of the relevant figures (Fig. 5 and Supplementary Figs 4–6). For simulating synergic interactions we started by randomly choosing multiple points as nucleation sites for molecular clusters of a reference species (green). We then distributed ‘reference molecules' (green molecules in panel a of Fig. 5 and Supplementary Figs 4–6) around the points that served as nucleation sites with a Gaussian clustering statistics (see Supplementary Methods for further details). ‘Directly recruited molecules' were simulated by distributing molecular species (red and blue molecules in Fig. 5a) around molecules from the reference species (again, green molecules in Fig. 5a). Thus, the simulated patterns of molecular arrangements (Fig. 5 and Supplementary Figs 4–6; B,H) are the result and manifestation of multiple, inter-related clustering processes. The results for all possible scenarios are summarized in Supplementary Table 2. We focus below on two important simulated cases, one is the only scenario that consistently shows positive interaction synergy (case 3 in Supplementary Table 2) and the other is an example of a scenario showing negative interaction synergy (case 7 in Supplementary Table 2).

### The case of positive interaction synergy

In the case of positive interaction synergy (Fig. 5a–f; case 3 in Supplementary Table 2), we considered a scenario where two types of molecules (red and blue) were recruited to the reference species (green molecules). However, one of the molecules (red) was recruited directly to the other (blue) molecules and was, therefore, indirectly brought close to the reference molecules (Fig. 5a–c). We regard this example as a positive case of molecular interaction synergy, as the recruitment of red molecules to green molecules was promoted by the presence of blue molecules. Indeed, our interaction synergy statistics accurately captured significant synergy between green and blue molecules in their recruitment of red molecules, as indicated by the PCFs (Fig. 5d,e). The length scale where synergy was significant corresponded to the effective range over which clustering and synergic interactions were simulated (see arrowheads in Fig. 5d,e and Supplementary Information for further details). Note that we show here both the conditional PCF g_12|Pr(2,3)_ (Fig. 5d) and its standardized form ĝ_12|Pr(2,3)_ (Fig. 5e), while earlier we showed only the standardized form in Figs 3 and 4 for brevity (see definitions in equations (6)–(10) in Supplementary Methods). The reason for now showing both is that the conditional PCFs capture the spatial organization (that is, clustering) of the interacting species as well as their synergy in each cell, but they cannot be averaged over multiple cells. In contrast, the standardized curves allow for their averaging over multiple cells, but factor out the extent of clustering of the interacting species in each cell. Both forms show significant interaction synergy indicated with a black arrowhead on the figure (Fig. 5d,e). The last panel (Fig. 5f) shows a cartoon of the interpretation of the interaction synergy analysis: red molecules are brought into the proximity of green molecules via interactions with blue molecules (as indicated by the curved arrow).

### An example of a case that shows no interaction synergy

As discussed earlier, there are multiple cases that do not constitute synergy in molecular interactions. All possible ‘negative' cases (with green molecules as a reference species) are shown in Fig. 5g–l and Supplementary Figs 4–6, summarized in Supplementary Table 2, and further discussed in Supplementary Methods (section on interaction synergy simulations). As an example, we focus here on a ‘negative' case with molecular interactions, but no synergy in these interactions (Fig. 5g–l; case 7 in Supplementary Table 2). In this specific example, red molecules were directly recruited to green molecules, but the location of blue molecules was independent of any other molecular species. As expected, the interaction synergy statistics indicated no significant synergy in the interaction of green and blue molecules with respect to red molecules (Fig. 5j,k). However, the upturn in the shape of the conditional PCF g_12|Pr(2,3)_ at small distances correctly indicated an interaction between red and green molecules (indicated by open arrowheads in Fig. 5j and Supplementary Fig. 4D,J). When there are no molecular interactions, the conditional PCF remains flat and horizontal as can be seen in panels D and J of Supplementary Figs 5 and 6. The interpretation of this analysis is shown in the cartoon as a single straight arrow representing only a direct interaction between green and red molecules with no synergy in the interaction (Fig. 5l). Multiple intricate scenarios of interactions between molecules can be considered and simulated (as detailed in Supplementary Table 2). For many of these cases we demonstrate that the shape of the non-standardized curves g_12|Pr(2,3)_ (height above baseline; panels d and j in Fig. 5 and Supplementary Figs 4–6) carry additional information in respect to the standardized curve, and that it correctly captured interactions of the relevant molecules (compare d and j in Fig. 5 and Supplementary Fig. 4 with panels D and J of Supplementary Figs 5 and 6; that is, cases 3, 7, 1, 2 versus cases 4, 5, 6, 8 in Supplementary Table 2). See further discussion on the classification of negative cases in Supplementary Information (section on interaction synergy simulations). We also discuss in Supplementary Information conditions where residual synergic correlation caused by a high density of randomly distributed molecules could in principle might be observed where it does not really exist (for example, for case 5 is shown in Supplementary Fig. 7). However, such cases are irrelevant to the experimental data in this study for the following reasons. First, these cases can be easily detected by the flat univariate of the randomly distributed species and did not appear in our experimental data or in previous studies^10^,^22^. Second, even if these cases exist, they do not affect the synergy analyses when the dense species is used as the reference molecule, as we have done in our experiments (see Supplementary Fig. 8 and discussion in Supplementary Information). Of note is that throughout the simulated cases, our statistics were aimed at the correct identification of synergic recruitment of blue molecules, via their interactions with red molecules, to green molecules. Alternative synergic interactions (for example, the recruitment of blue molecules via red molecules to green molecules, described in Supplementary Fig. 4G–L) were correctly identified by related PCFs (for example, g_13|Pr(2,3)_ and ĝ_13|Pr(2,3)_), but not shown for brevity (again, see further discussion in the section on interaction synergy simulations of Supplementary Information).

Generally, multiple interaction scenarios can act simultaneously between the same species. In such cases, our statistics would average out the diversity of molecular interactions and would likely not be able to resolve the different interaction scenarios. Nevertheless, from the set of well-defined scenarios we simulated, we conclude that our analyses could capture synergy in molecular interactions correctly where it existed, while excluding synergy in molecular interactions where it did not play a role.

### Robustness against experimental limitations and artefacts

The second major issue is that various experimental limitations and artefacts could further affect the robustness of the measures of interaction synergy and localization topology. One such artefact might be due to the cross-talk between the imaged channels. To check the sensitivity of our synergy analyses to this effect, we conducted simulations of all cases described in Supplementary Table 2 with the addition of cross-talk between the imaging channels. Specifically, to control for this effect, we considered a range of cross-talks from the ‘blue' to the ‘green' channel, ranging from 0 to 30% (compare with the evidence for 1–2% cross-talk in Fig. 1a,b). We noticed that most cases were unaffected by cross-talks as high as 30%. However, case 6 (where no interaction synergy is expected; see Supplementary Table 2) indicated interaction synergy with a cross-talk as low as 10% (as shown in Supplementary Fig. 9A–F). This is understandable since some of the ‘blue' molecules become ‘green' and make this case effectively similar to case 3 (Fig. 5a). Considering cross-talk from the ‘green' to the ‘blue' channel gave similar results. Most simulated cases seemed insensitive to cross-talks ranging from 0 to 30% (Supplementary Fig. 9G), while cases 1 and 4 (see Supplementary Table 2) yielded on some occasions positive interaction synergy, starting at 10% cross-talk (Supplementary Fig. 9G). This occurred in fewer than 10% of the simulations for these cases. Nonetheless, these results emphasize the need for a low cross-talk (<10%) between the imaged channels for our analyses, which we were able to achieve in our study.

Other artefacts might include random undersampling of the detected molecules due to the existence of a population of endogenous, unlabelled proteins, or due to incomplete detection of labelled proteins. Therefore, we checked the robustness of the interaction synergy analysis to undersampling by focusing on the case of positive interaction synergy (presented in Fig. 5a–f), then randomly selecting a subset of one of the molecular species (Supplementary Fig. 10A–C) and monitoring the effect of selection on the resulting statistics (Supplementary Fig. 10D,E). Considering >60% type 3 (Red) molecules did not affect the interaction synergy measure. Considering 40% of the molecules partially diminished the significance of the measure, while considering only 20% of the molecules often eliminated the significance (Supplementary Fig. 10D).

Alternatively, experimental limitations could lead to the over-sampling of fluorophores and, thus, to the over-counting of the molecules that they tag. Indeed, some useful fluorophores for super-resolution imaging might reactivate (for example, Dronpa^17^) or blink (for example, synthetic fluorophores used in dSTORM). If the statistics of such reactivation and blinking effects are not known or improperly accounted for when attempting to count molecules, detected molecules might be over-sampled (that is, counted more than once). Such over-sampling would result in an apparent aggregated (or clustered) state of the relevant molecular species under study. In addition, concerns have been raised regarding the self-aggregation of FPs (for PALM^34^) and primary and secondary staining antibodies (for dSTORM^35^). Again, such artefactual aggregation could be falsely interpreted as molecular clustering and affect our interaction synergy and localization topology statistics. Since both over-counting and artefactual aggregation produce similar patterns of over-clustered molecules, we examined the robustness of our statistical statistics to these effects with the same set of simulations (focusing again on the case of positive interaction synergy presented in Fig. 5a–f). We first randomly under-sampled one of the molecular species. We then randomly added molecules in close proximity to the remaining molecules of the same species until we reached a target over-sampling factor. Next, we calculated the interaction synergy statistics and interpreted their result as in the previous case of under-sampling. Here we found that over-sampling (up to a factor of ∼3, Supplementary Fig. 10F–H) did not affect the interaction synergy analysis (Supplementary Fig. 10I–J).

The same simulations can be used to test the robustness of the topology measure (as in Fig. 2d) to these artefacts. In the case of under-sampling of molecules, we found that the topology measure could properly detect the localization hierarchy of molecular species when only 40% of the molecules were used in the analysis (Supplementary Fig. 11A). When the level of detection was only 20% of the molecules, the topology measure incorrectly reported that the red molecules were closer to blue molecules (Supplementary Fig. 11A). In the simulations of over-sampling, the topology measure correctly predicted the relative localization hierarchy between red, green and blue molecules even when over-sampled by threefold (Supplementary Fig. 11B).

Next, we tested the effect of experimental errors in the localization of molecules on the localization topology and interaction synergy measures. We simulated molecular patterns according to case of positive interaction synergy (case 3 in Supplementary Table 2), described in Fig. 5a–f. Then, we simulated 20 realizations where we introduced random errors (drawn from a Gaussian distribution with 1σ matching the experimental errors) to the localizations of all molecules. The results in Supplementary Fig. 12A indicated the correct localization hierarchy of the different species with little effect of the localization errors on the localization topology values. We also wanted to test here whether the choice of the molecular species that serves as reference for clustering influenced the localization hierarchy. That is, would it make a difference if the clusters were not centred on LAT, but rather on one of the other molecules found in the signalling complexes. Again using the example of the case of positive interaction synergy, we chose in each case a different molecular species as a reference for clustering (red, green or blue). The chosen reference species had always a localization topology value of 1 (see further details on recruitment statistics in Supplementary Methods). The results in Supplementary Fig. 12A,B indicated the correct localization hierarchy of the different species in all cases. Note that the absolute values of the localization topology for each case were expectedly affected by the choice of the reference molecular species. The correct hierarchy for the simulated data here should be blue in the centre of clusters with the highest localization topology value, with green in the middle and having intermediate localization topology value, and with red farthest from the centre of the clusters with the lowest localization value. To conclude, the shown data accounts for the effects of both localization errors (Supplementary Fig. 12A) and the choice of reference species on the topology analyses (Supplementary Fig. 12B). Finally, we quantified the effect of the localization errors on the interaction synergy analysis. Thus, we calculated the average of the 20 standardized conditional PCFs for the 20 simulated realizations that included the localization errors. Remarkably, the interaction synergy curve (Supplementary Fig. 12C) was little affected by the localization errors (error-bars indicate 2 s.e.m.), and indicated significant positive interaction synergy, as expected (compare the interaction synergy results of the simulated localization errors with the results of the ground truth shown in Fig. 5e and in Supplementary Fig. 12C, for convenience).

## Discussion

Here, we developed and applied MC-PALM to study properties that cannot be directly accessed by current biochemical and imaging techniques including the multimolecular interactions, nanoscale organization and content of signalling complexes at the single-molecule level. We first validated our technique by imaging three different proteins, actin, LAT and TCR, at the surface of fixed and live T cells. To accomplish this we used a new approach to multicolour PALM imaging by which three protein species bearing three different PAFP tags can be detected in only two spectral imaging channels, with minimal (<2%) cross-talk (that is, species misidentification). We then applied our imaging technique to resolve the nanoscale organization of LAT-nucleated signalling complexes in activated T cells. LAT is a critical adapter protein that nucleates binding of multiple adapter and effector molecules upon TCR stimulation, thus regulating multiple pathways in activated T cells^14^. We recently discovered that LAT-nucleated signalling complexes have nanoscale structure, as SLP-76 localized to the periphery of LAT nanoclusters^10^. In a major expansion of our previous work^10^,^22^, here we introduce statistical measures to describe the hierarchical organization of molecules in clusters in a precise, non-subjective and automated way.

Notably, our analyses of the localization topology and of the synergy of molecular interactions are applicable to various single-molecule multicolour imaging modalities^36^ (for example, based on the super-resolution techniques of FPALM, STORM, dSTORM, GSDIM and Immunogold labelling TEM) and to any type of second-order bivariate statistics (including Ripley's K- and L-statistics). For that, we provide a detailed account and guidelines for the choice of various parameters (for example, thresholds) involved in our statistics in Supplementary Information (Analyses section). To validate the robustness and wide applicability of our statistical measures, we turned to simulations of all possible molecular interaction scenarios (with a specific molecule as a reference species) and of various experimental artefacts that are commonly involved in single molecule super-resolution imaging. Indeed, we have determined that our statistics will show the correct interaction synergy and hierarchy of molecular clustering for the simulated scenarios and in the face of multiple artefacts. Such artefacts include cross-talk between imaging channels of up to 30%, over-sampling by up to threefold, under-sampling by consideration of down to 40% of the molecules or mislocalizing the molecules by typical experimental errors. We have also shown that the correct hierarchy will be described even if we choose any of the molecule species within a cluster as a reference molecule for the measure of localization topology.

Using MC-PALM and localization topology statistics, we were able to resolve the structure of LAT nanoclusters in molecular detail in three colours. Specifically, our measure of localization topology aims to highlight that some species are recruited to the centre of clusters, while others tend to localize to the periphery of clusters (that is, forming a ring) independent of the exact morphology of the clusters. We observed that VAV1 and PLCγ1 localized well within LAT clusters with high measures of localization topology (Fig. 2). In contrast, SLP-76 and actin showed significantly lower levels of localization topology (Fig. 4e). Our analyses indicated concentric arrangements of molecules at LAT clusters, with VAV1 and PLCγ1 at the centre, SLP-76 towards the periphery and actin surrounding the clusters.

In the context of molecular complexes, and in particular, in the signalling complexes studied here, positive or negative cooperativity can be broadly defined as the increase or decrease, respectively, in the probability of finding a specific molecular interaction in the presence of a third molecular species. Indeed, cooperativity has been suggested to play an important role in the recruitment of signalling proteins to multimolecular complexes^18^,^32^,^37^. The methods we have developed for imaging and analysis enabled us to study trivariate relationships between three individual proteins within signalling complexes at the PM of activated cells. Importantly, our imaging method does not report on immediate molecular binding events. Thus, we depend on previous biochemical and FRET data^18^,^30^,^31^ to interpret our data in terms of direct binding events and cooperativity in molecular interactions.

**Figure 6:**
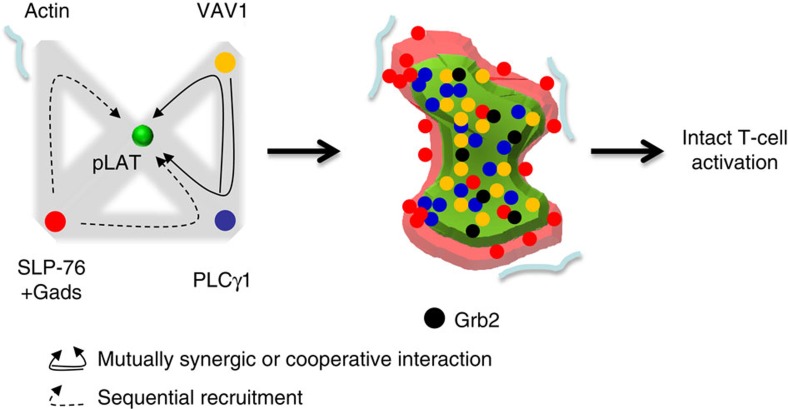
A model of sequential and hierarchical recruitment of effectors to LAT clusters. A model that summarizes the set of hierarchical molecular interactions (left) that lead to the hierarchical structure (right) of LAT-nucleated clusters and intact T-cell activation. PLCγ1 and VAV1 show mutual interaction synergy, interpreted here as cooperativity, in their recruitment to LAT clusters. SLP-76 molecules in proximity to PLCγ1 or actin showed enhanced proximity to LAT molecules, but proximity to SLP-76 did not increase the recruitment of PLCγ1 or VAV to LAT. The clusters are ordered such that VAV1 molecules are closest to LAT in the centre of the cluster, PLCγ1 molecules are the next closest, with most SLP-76 and actin molecules around the periphery.

Our results reveal an intricate and hierarchical network of synergic (interpreted here as cooperative) interactions that facilitate the formation of LAT-nucleated signalling complexes (Fig. 6). Significant and mutual synergy (or cooperativity) was found for VAV1 and PLCγ1 as they bind LAT. Surprisingly, however, the interactions of PLCγ1 and SLP-76 or actin and SLP-76, were not mutual with respect to LAT clusters. The recruitment of subsets of SLP-76 molecules to LAT clusters was promoted by interactions with PLCγ1 and actin. However, both the PLCγ1 and actin associations with LAT clusters were independent of SLP-76. These results cannot be explained by previous models that suggested that LAT-bound Gads-SLP-76 complexes nucleate actin polymerization^30^,^32^ or that mutual cooperativity between SLP-76 and PLCγ1 stabilize PLCγ1 binding to LAT^18^.

Previous studies have shown striking dynamics of molecules at the evolving IS. Actin has been shown to colocalize with activated TCR and LAT at the early lamellar contacts of a spreading T cell^30^. Later, actin clears quickly from the centre of the interface to form a ring surrounding the IS^38^. Also, upon TCR triggering, SLP-76 has been shown to get quickly recruited to phosphorylated tyroines on LAT through its constitutive interaction with Gads, and has been suggested to nucleate actin polymerization^30^. However, SLP-76 clusters seem to separate from LAT clusters and translocate to the centre of the IS^39^. In this study we observed that within a minute of the initial activation of the TCR and before extensive cell spreading and actin clearing, polymerized actin surrounds LAT clusters and does not require SLP-76 to maintain this pattern. To interpret our unexpected results and relate them to previous studies, we propose a model of a sequential and hierarchical mechanism for the formation of LAT signalling clusters. We conjecture that polymerized actin supports the nucleation of LAT clusters and SLP-76 complexes, helps to shape the PM and possibly traps LAT clusters within transient actin corrals^40^. An earlier recruitment of actin to SLP-76 complexes on phosphorylated LAT is still possible but must be highly transient^30^,^32^. Coincident with the early nucleation process, the recruitment of Gads-bound SLP-76 to the distal phophotyrosines of LAT at the periphery of LAT clusters is facilitated by the recruitment of PLCγ1 to LAT and the presence of proximal actin. The interactions of LAT, SLP-76 and actin are transient, as actin clears from the centre of the T-cell interface and SLP-76 translocates to the centre of the interface.

Taken together, our methods and results shed light on the complex and hierarchical process of nucleation of LAT complexes and clusters. The combination of the techniques described herein can aid in the study of various macromolecular structures by super-resolution microscopic methods in many different biological systems. Better understanding of the nanoscale organization and cooperativity of molecular interactions within signalling complexes could reveal novel ways of intervening in critical cellular mechanisms of cell activation.

## Methods

We provide below a brief description of the materials and methods, and a complete description in Supplementary Methods.

### Samples

Proteins tagged with the PAFPs Dronpa (MBL International Corporation), PAmCherry and PAGFP were generated in EGFP-N1 or EGFP-C1 vectors (Clontech). Specifically, newly generated constructs for this study included PLCγ1-Dronpa, PLCγ1-PAGFP, PAGFP-Actin, PAmCherry-Actin, Dronpa-Actin, VAV1-PAmCherry, TCRζ-PAGFP and SLP-76-PAGFP. Validation of cloning was done by restriction digestion analyses and DNA sequencing of the inserts. E6.1 Jurkat T cells were transfected with DNA using a nucleofector shuttle system, program H-10 and the Amaxa T-kit (Lonza). Transiently transfected cells were maintained in transfection medium, sorted for positive expression of PAmCherry, Dronpa or PAGFP chimeras and imaged within 48–72 h from transfection. Stable cell lines were created by selection with Geneticin at 1.5 mg ml^−1^ (G418, Invitrogen) and checked by flow cytometry for positive expression of fluorescently tagged proteins. Cells were then evaluated using biochemistry assays, flow cytometry, confocal microscopy (510 LSCM, Zeiss) and epifluorescence, TIRF and PALM imaging, as described below. The preparation of coverslips (#1.5 glass chambers, LabTek) for imaging spread cells followed a previously described technique^20^. These coverslips were then coated with 100-nm gold beads (Microspheres-nanospheres) that had been sonicated and diluted 10 × in methanol. Throughout the study we used the following antibodies that were validated in previous studies^7^,^10^: A purified mouse αhuman αCD3 (clone Ucht1) served as a TCR stimulatory antibody that caused robust T-cell activation and spreading. An αCD45 antibody (BD Biosciences) was used as a non-stimulatory antibody that lead to T-cell spreading without TCR triggering and cell activation. Cleaned coverslips with beads were incubated with stimulatory or non-stimulatory antibodies at a concentration of 10 μg ml^−1^ overnight at 4 °C or 2 h at 37 °C. Finally, chambers and coverslips were washed with PBS. A few hours before imaging, cells were resuspended in imaging buffer at a concentration of 1 million per 150 μl and 100,000–500,000 cells were dropped onto coverslips for PALM or diffraction limited imaging, incubated at 37 °C for the specific spreading time (typically 3 min) and fixed with 2.4% PFA for 30 min at 37 °C.

### Imaging

Confocal imaging was performed using a 510 LSCM confocal microscope using a × 63, 1.4 NA objective (Zeiss). MC-PALM imaging was conducted similarly to the imaging previously described^10^, using a TIRF Nikon microscope. However, here the imaging sequence of tagged proteins followed the sequence described in Supplementary Table 1. As a first step, Dronpa-tagged proteins were imaged using continuous and low-intensity ∼340 nm illumination of an arc lamp (DAPI cube) and laser excitation at 488 nm in TIRF mode. Dronpa-tagged molecules were imaged for 10 s for live-cell imaging, or the depletion of their emission, as identified by the loss of fluorescence, for fixed-cell imaging. For cells expressing higher levels of Dronpa-conjugated proteins, it was critical to remove as many Dronpa molecules as possible to avoid bleedthrough in the PAGFP images. The focus of the microscope was then adjusted using the PerfectFocus system of the microscope. Similar focus adjustments were also performed after each imaging step that followed, as described below. After imaging Dronpa, the sample was illuminated with maximal intensity of the Arc lamp illumination at ∼440 nm (CFP cube) for 10 s for imaging living cells and 10–20 s for imaging fixed cells. This step served to activate PAmcherry- and PAGFP-tagged proteins and to photobleach residual Dronpa-tagged proteins. Longer activation times are more efficient at photobleaching Dronpa. We next imaged PAmCherry-tagged proteins, following by imaging PAGFP-tagged proteins. Each imaging step took 10 s for live-cell imaging and ∼30 s for fixed cells. The sequence of steps of photoactivation, imaging PAmCherry and PAGFP were then repeated multiple times: six times in total for live-cell imaging or until depletion of emission from all molecules for fixed-cell imaging.

As with other imaging technique, our MC-PALM approach requires the complexes under study to be relatively stable through the effective frame time of imaging (∼20–30 s). Shortening the acquisition time could be achieved by using faster cameras, brighter fluorophores, enhancing excitation of fluorophores using brighter lasers, and using algorithms that can detect molecules with over-lapping point-spread functions (for example, ref. 41). Also, it should be noted that Dronpa photobleaching is essentially accomplished already at the beginning of the imaging sequence and that its fluorescence decays fast and exponentially upon photobleaching (with a lifetime *τ*<2 s). Thus, Dronpa photobleaching could be shortened to further accelerate imaging, esp. after the first cycle of imaging. Throughout imaging, 100-nm gold beads (Microspheres-Nanospheres) were used as fiduciary markers to account for drift and for registration of the MC-PALM channels. Typical registration between the MC-PALM channels was <10 nm across the imaging field. Sample sizes were chosen to account for cell to cell variability, within experimental constraints. Notably, each measurement of individual cells contained tens to hundreds of molecular clusters, over which the localization topology and interaction synergy statistics were calculated (as detailed below).

### Analyses

Movies generated by MC-PALM imaging were analysed by the PeakSelector software 5 for the identification of individual peaks in the movie frames. Next, peaks were grouped and assigned to individual molecules for rendering of the MC-PALM images. Peak grouping used a distance threshold and a temporal gap to account for possible molecular blinking^42^. A range of temporal gaps were considered for each fluorophore separately in order to minimize possible over-counting of molecules. Individual molecules were presented in MC-PALM images with intensities that correspond to the probability density values of their fitted Gaussian with respect to the maximal probability density values detected in the field. This scale was set for each species separately for proper appearance in the PALM images and should not be interpreted as the density (or number) of molecules in clusters.

The localization topology and interaction synergy simulations are detailed in Supplementary Information due to the length of these subsections.

### Data availability

Data supporting the findings of this study are available within the article and its Supplementary Information files and from the corresponding author upon reasonable request.

## Additional information

**How to cite this article:** Sherman, E. *et al.* Hierarchical nanostructure and synergy of multimolecular signalling complexes. *Nat. Commun.* 7:12161 doi: 10.1038/ncomms12161 (2016).

## Supplementary Material

Supplementary Information Supplementary Figures 1-12, Supplementary Tables 1-2, Supplementary Methods and Supplementary References
