# Supplementary material for: Hierarchical nanostructure and synergy of multimolecular signalling complexes
Source: Nat Commun. 2016 Jul 11;7:12161. doi: 10.1038/ncomms12161 (PMC4942584; doi:10.1038/ncomms12161)
Supplement: Supplementary Information — Supplementary Figures 1-12, Supplementary Tables 1-2, Supplementary Methods and Supplementary References [file ncomms12161-s1.pdf]

Supplementary Figure 1

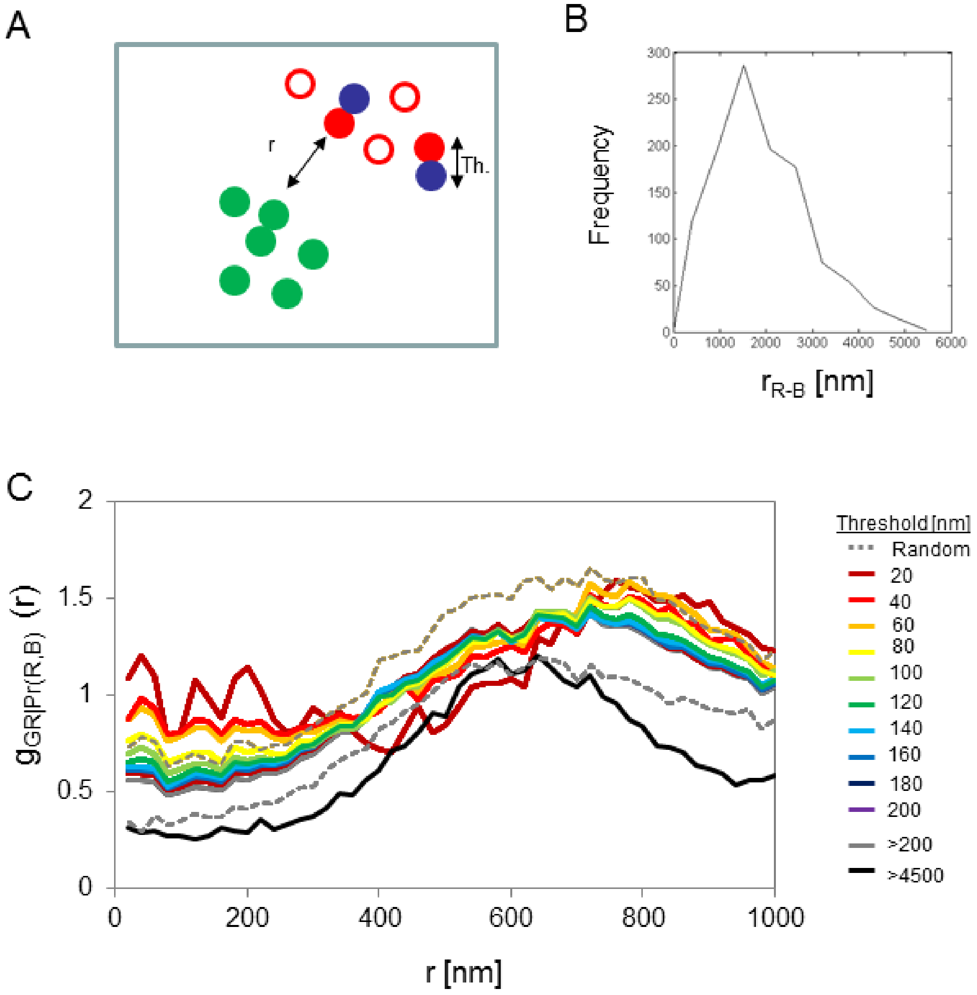

Supplementary Fig. 1. Robustness of interaction synergy analysis

(A) A scheme depicting the analyses of interaction synergy between molecules of three types. Here red molecules are recruited to green molecules through binding to blue molecules. Red molecules are selected based on their proximity to blue molecules (red filled circles mark red molecules found below a threshold distance, Th., from blue filled circles). Bivariate PCF analysis is then conducted between the selected subset of red molecules from green molecules. The distances, r, marks the length-scale of the interaction between the red and green molecules. (B) The distribution of distances between red and blue molecules in a representative test sample of cells presented in Fig. 4A that expressed PLCγ1-PAGFP, LAT-Dronpa and SLP-76-PAmCherry (corresponding to the blue, green and red molecules, respectively). (C) Conditional bivariate PCFs, analyzing the synergy of binding of red and green molecules upon the binding of red molecules to blue molecules. Each curve describes a different threshold for the determination of proximity between red and blue molecules (bold colored lines) or the exclusion of such molecules (grey and black bold lines). The results are compared to a 95% confidence interval due to non-synergic interactions between red and green molecules (gray dotted lines; see Analyses section in SI for further details). Here, the presented conditional bivariate PCFs are shown for a single cell and were not standardized. Otherwise, all conditional bivariate PCFs throughout the text were calculated with a proximity threshold of 40 nm, standardized and averaged for multiple cells (for example, compare this panel with Fig. 4C; see further details in the Analyses part of the SI).

Supplementary Figure 2

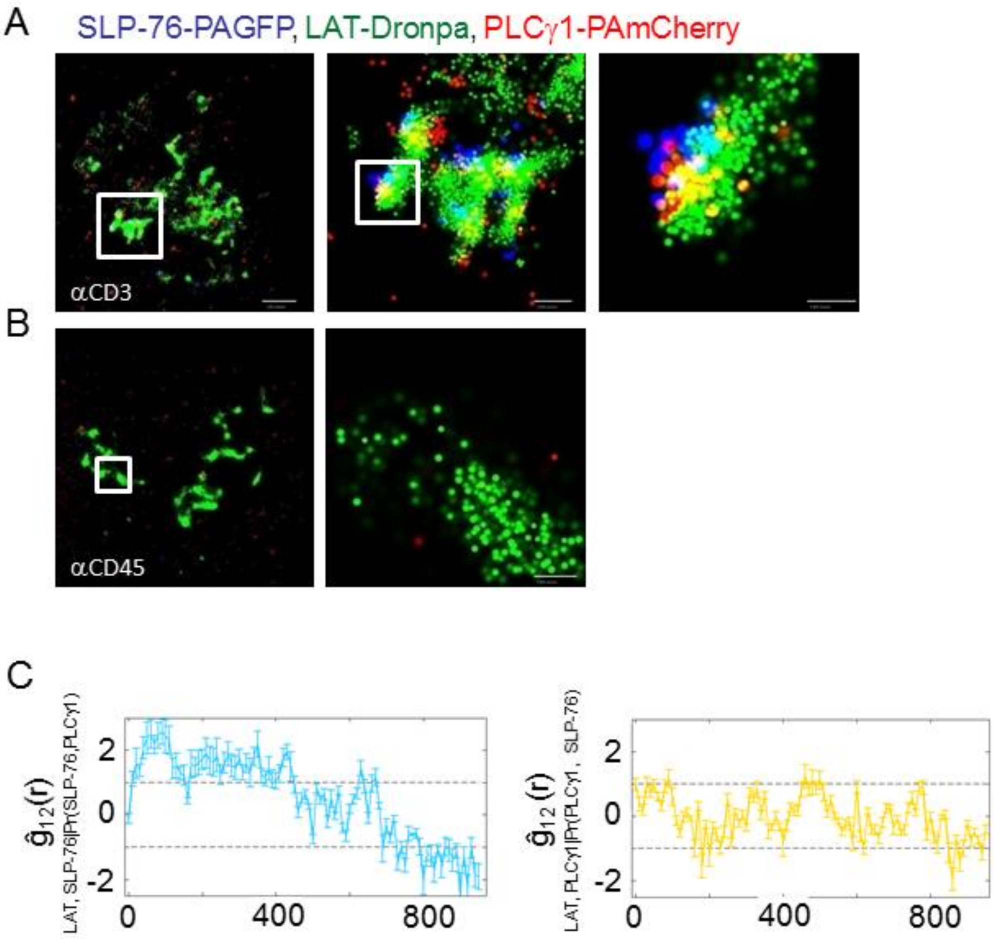

Supplementary Fig. 2. Observed patterns for SLP-76, LAT and PLCγ1 are insensitive to color reversal

MC-PALM imaging of fixed Jurkat cells expressing SLP-76-PAGFP (blue), LAT-Dronpa (green) and PLCγ1-PAmCherry (red) spread for 3 min on (A) αCD3-coated or (B) αCD45-coated coverslips. Zoomed images of representative LAT clusters are shown. Maximal probability density values for PAGFP, Dronpa and PAmCherry rendering – (A) 200, 310, 250 molecules per μm^2, respectively. Bars – 2 μm (left), 500 nm (middle) and 200 nm (right). (B) 300, 250, 300 molecules per μm^2, respectively. Bars – 2 μm (left) and 200 nm (right). (C) A conditional bivariate PCF(standardized) analyzing the interaction synergy of SLP-76 and PLCγ1 on the binding of SLP-76 to LAT (left) or the interaction synergy of PLCγ1 and SLP-76 on the binding of PLCγ1 to LAT (right). Compare these results with the conditional bivariate PCF curves in Fig. 4C. Error bars are SEM.

Supplementary Figure 3

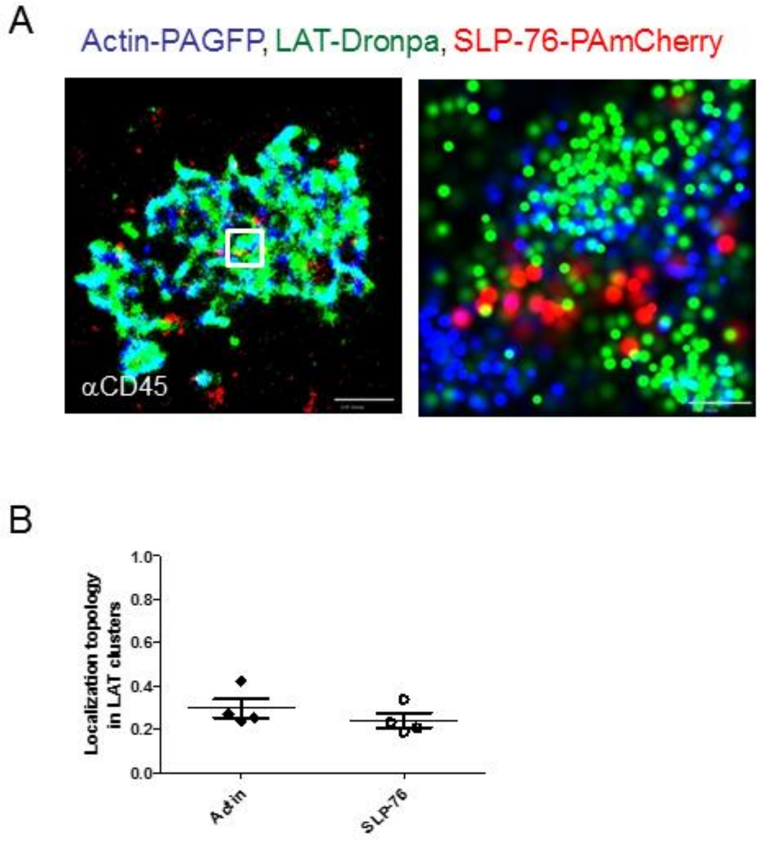

Supplementary Fig. 3. Pattening of actin, LAT and SLP-76 in non-activated cells

(A) MC-PALM imaging of fixed Jurkat cells expressing Actin-PAGFP (blue), LAT-Dronpa (green) and SLP-76-PAmCherry (red), spread on αCD45-coated coverslips for 3 min. Zoomed image of representative LAT clusters is shown. Maximal probability density values for PAGFP, Dronpa and PAmCherry rendering – (A) 340, 290, 320 molecules per μm^2, respectively. Bars – 2 μm (left) and 200 nm (right). (B) Relative localization topologies for the expressed molecules in multiple cells as in panel A. Error bars are SEM.

Supplementary Figure 4

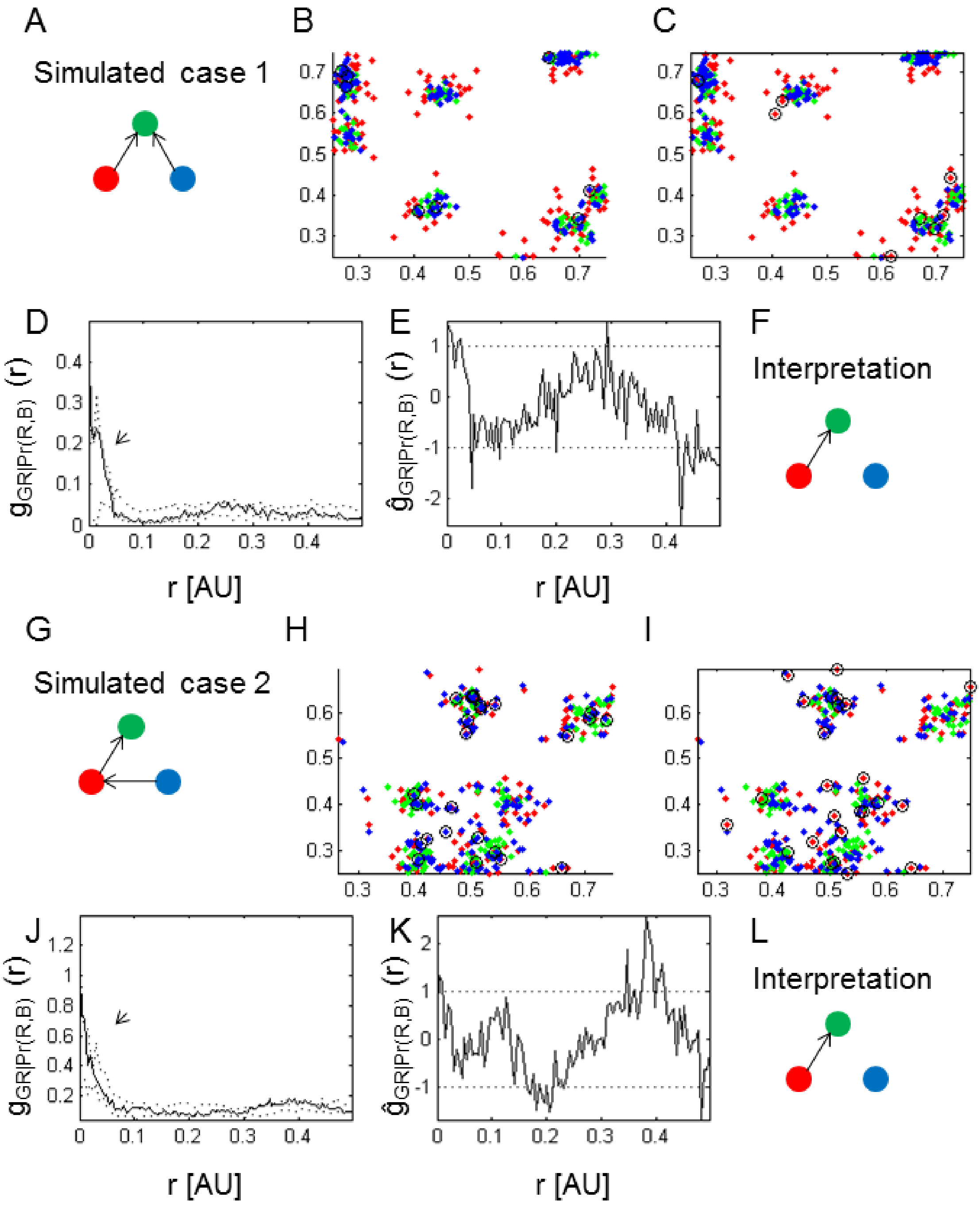

Supplementary Fig. 4. The interaction synergy analysis distinguishes additional cases of non-synergic molecular interactions (cases 1-2)

Red molecules are selected based on their proximity to blue molecules (red filled circles). Bivariate PCF analysis is then conducted between the selected subset of red molecules and green molecules.

(A-F) Case 1 with no interaction synergy: Simulated data and interaction synergy analyses of a case depicted in panel A. (A) The model of molecular interactions where red and blue molecules were directly recruited to the reference green molecules. (B) A representative simulated data (n = 20). (C) A representative view of molecular distributions from one of nineteen Monte-Carlo simulations where where red molecules were selected randomly. (D) The conditional bivariate PCF of the simulated data in panel B, where dotted lines mark the 95% confidence interval due to the Monte-Carlo sets (as shown in panel C). Open arrow-head indicates significant upwards turn of the conditional PCF curve, indicating correlation of the green and red species. (E) The standardized conditional PCF (i.e. the standardized form of the curve in panel D). (F) The resultant molecular interaction scheme based on the interpretation of the $g_{GR|Pr(R,B)}$ and $\hat{g}_{GR|Pr(R,B)}$ statistics in panels D and E. Direct recruitment of red molecules to green molecules is marked with a straight arrow between the interacting species.

(G-L) Case 2 with no interaction synergy: Simulated data and interaction synergy analyses of a case depicted in panel G, where no interaction synergy applies. The data in these panels is arranged as in panels A-F of this figure and are briefly described below. (G) The model of molecular interactions, where red molecules were recruited to the reference green species and blue molecules were recruited to red molecules (compare this scheme with the molecular interaction scheme of Fig. 5G and Supplementary Fig. 4A). (H) A representative

simulated data (n = 20). (I) A representative view of molecular distributions from one of nineteen Monte-Carlo simulations where red molecules were selected randomly. (J) The conditional bivariate PCF of the simulated data in panel H, where dotted lines mark the 95% confidence interval due to the Monte-Carlo sets (as shown in panel I). Open arrow-head indicates significant upwards turn of the conditional PCF curve, indicating correlation of the green and red species. (K) The standardized conditional PCF (i.e. the standardized form of the curve in panel J). (L) The resultant molecular interaction scheme based on the interpretation of the $g_{GR|Pr(R,B)}$ and $\hat{g}_{GR|Pr(R,B)}$ statistics in panels J and K. Direct recruitment of red molecules to green molecules is marked with a straight arrow between the interacting species.

Supplementary Figure 5

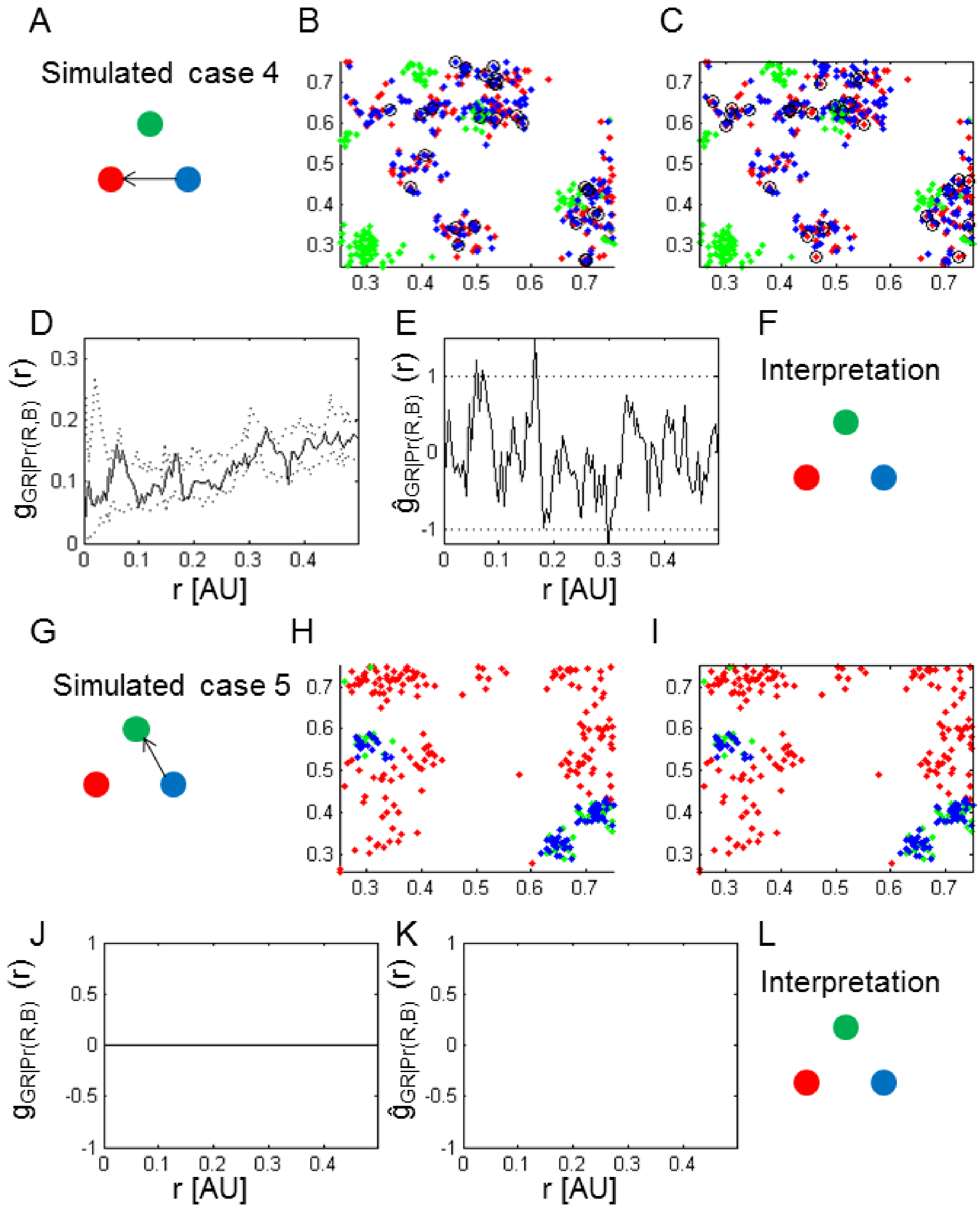

Supplementary Fig. 5. The interaction synergy analysis distinguishes additional cases of non-synergic molecular interactions (cases 4-5)

Red molecules are selected based on their proximity to blue molecules (red filled circles). Bivariate PCF analysis is then conducted between the selected subset of red molecules and green molecules.

(A-F) Case 4 with no interaction synergy: Simulated data and interaction synergy analyses of a case depicted in panel A. Panels A-F are as outlined in Supplementary Fig. 4. (A) The model of molecular interactions, where blue molecules were recruited to red molecules, but the placement of red molecules was unrelated to the placement of the reference green species. (B) A representative simulated data (n = 20). (C) A representative view of molecular distributions from one of nineteen Monte-Carlo simulations where red molecules were selected randomly. (D) The conditional bivariate PCF of the simulated data in panel B. (E) The standardized conditional PCF. (F) The resultant molecular interaction scheme. No interactions were identified for this case by the $g_{GR|Pr(R,B)}$ and $\hat{g}_{GR|Pr(R,B)}$ statistics in panels D and E.

(G-L) Case 5 with no interaction synergy: Simulated data and interaction synergy analyses of a case depicted in panel G. Panels G-L are as outlined in Supplementary Fig. 4. (G) The model of molecular interactions, where blue molecules were recruited directly to the reference green species, while the placement of red molecules was unrelated to the placement of the reference green species. (H) A representative simulated data (n = 20). (I) A representative view of molecular distributions from one of nineteen Monte-Carlo simulations where red molecules were selected randomly. (J) The conditional bivariate PCF of the simulated data in panel B. (K) The standardized conditional PCF. In this case, not enough red molecules are brought in close proximity to blue molecules (i.e. the operator

Pr(R,B) yields an empty group). Thus the conditional PCF in panel J is completely flat and the standardized conditional PCF in panel K cannot be calculated. (L) The resultant molecular interaction scheme. No interactions were identified for this case by the $g_{GR|Pr(R,B)}$ and $\hat{g}_{GR|Pr(R,B)}$ statistics in panels J and K.

Supplementary Figure 6

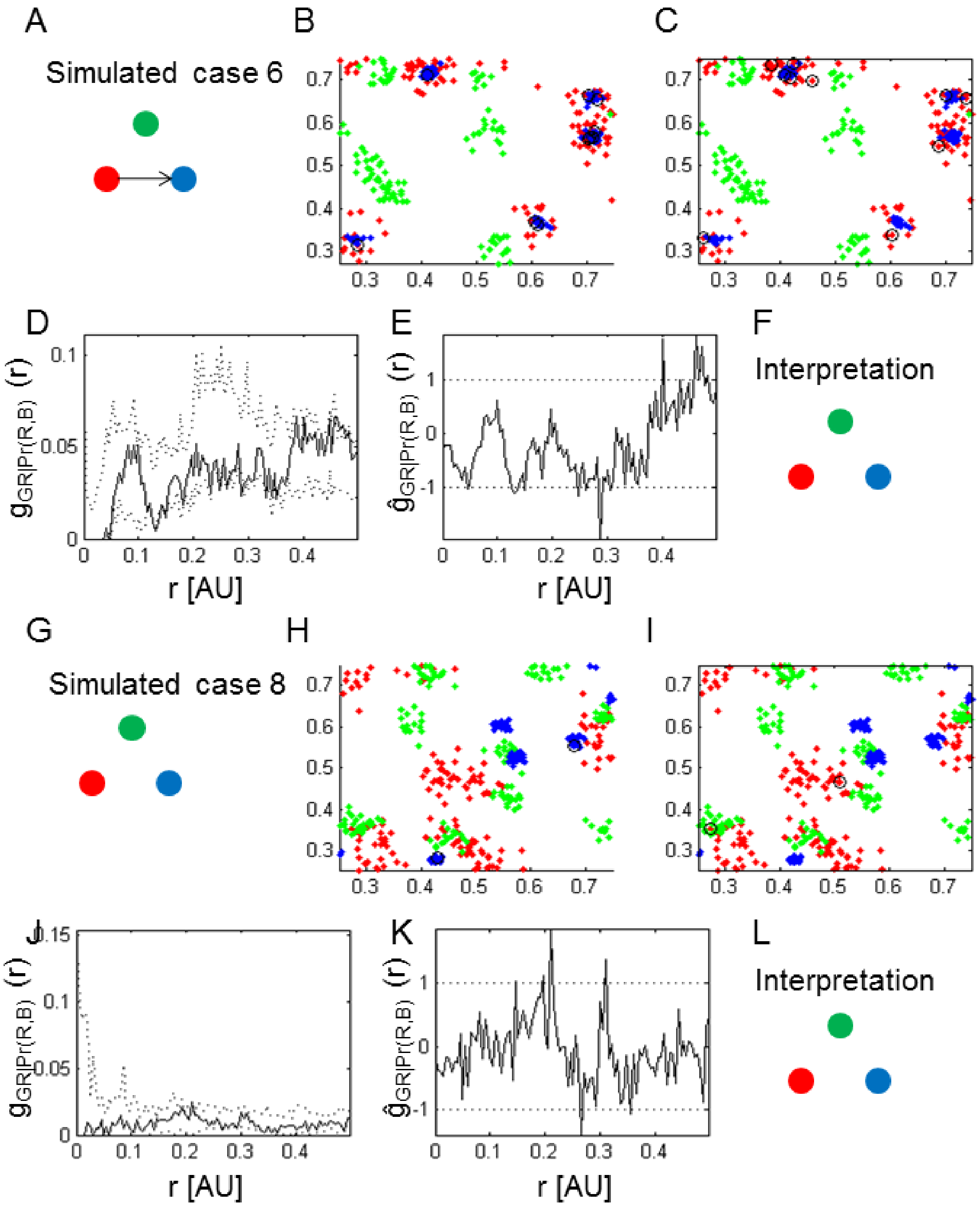

Supplementary Fig. 6. The interaction synergy analysis distinguishes additional cases of non-synergic molecular interactions (cases 6, 8)

Red molecules are selected based on their proximity to blue molecules (red filled circles). Bivariate PCF analysis is then conducted between the selected subset of red molecules and green molecules.

(A-F) Case 6 with no interaction synergy: Simulated data and interaction synergy analyses of a case depicted in panel A. Panels A-F are as outlined in Supplementary Fig. 4. (A) The model of molecular interactions, where red molecules were recruited to blue molecules, while the placement of blue molecules was unrelated to the placement of the reference green species. (B) A representative simulated data (n = 20). (C) A representative view of molecular distributions from one of nineteen Monte-Carlo simulations where red molecules were selected randomly. (D) The conditional bivariate PCF of the simulated data in panel B. (E) The standardized conditional PCF. (F) The resultant molecular interaction scheme. No interactions were identified for this case by the $g_{GR|Pr(R,B)}$ and $\hat{g}_{GR|Pr(R,B)}$ statistics in panels D and E.

(G-L) Case 8 with no interaction synergy: Simulated data and interaction synergy analyses of a case depicted in panel G. Panels G-L are as outlined in Supplementary Fig. 4. (G) The model of molecular interactions, where the placement of red, green and blue molecules were unrelated to each other. (H) A representative simulated data (n = 20). (I) A representative view of molecular distributions from one of nineteen Monte-Carlo simulations where red molecules were selected randomly. (J) The conditional bivariate PCF of the simulated data in panel B. (K) The standardized conditional PCF. (L) The resultant molecular interaction scheme. No interactions were identified for this case by the $g_{GR|Pr(R,B)}$ and $\hat{g}_{GR|Pr(R,B)}$ statistics in panels J and K.

Supplementary Figure 7

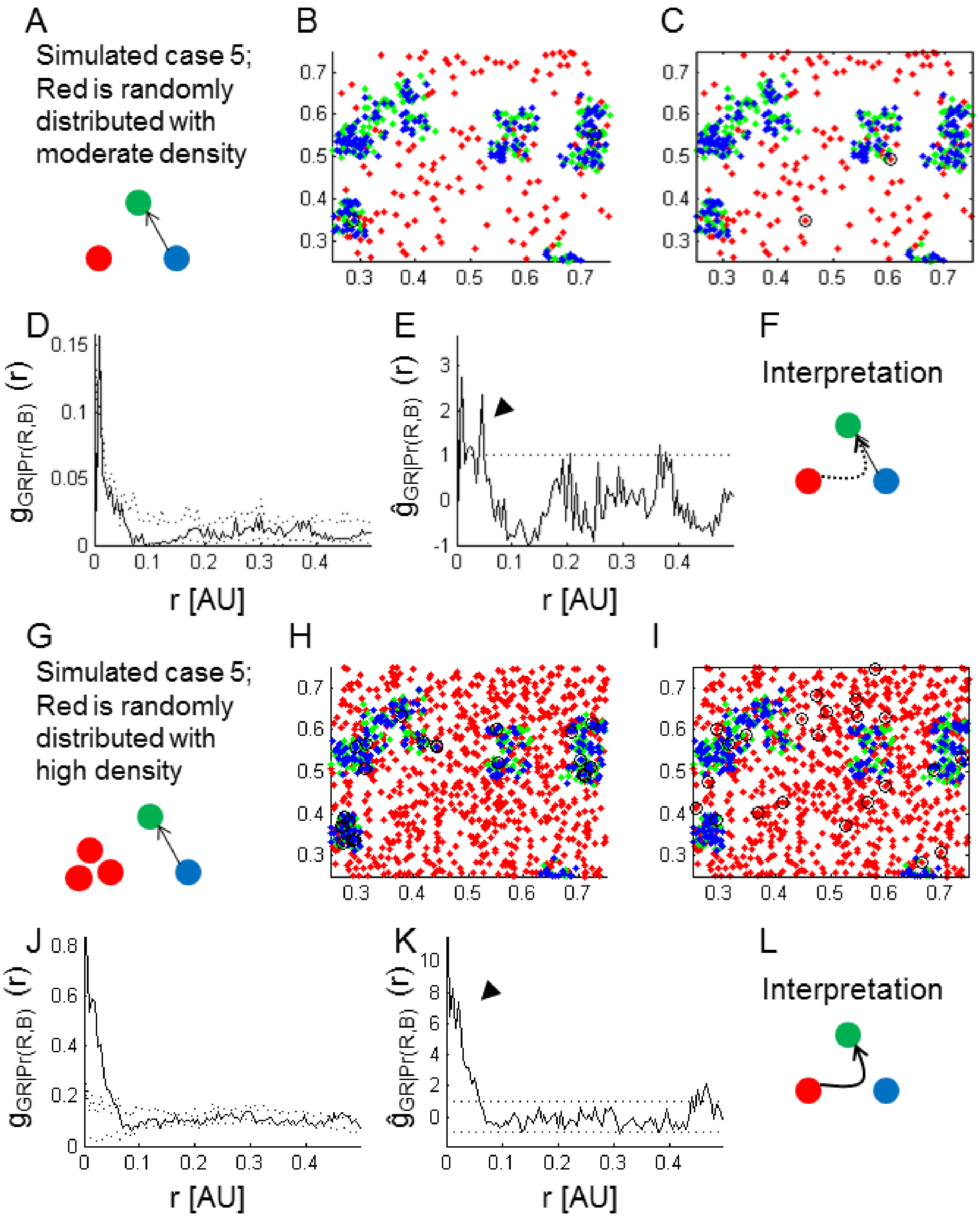

Supplementary Fig. 7. Random distribution of molecules beyond a threshold density may show 'apparent' synergy for non-synergic molecular interactions (case 5)

(A-F) Case 5 with no synergy where red molecules are distributed randomly with moderate density: Simulated data and synergy analyses of a case depicted in panel A. Panels A-F are as outlined in Supplementary Fig. 4. (A) The model of molecular interactions, where blue molecules were recruited to red molecules, but the placement of red molecules followed a Poisson distribution (and was thus also unrelated to the placement of the reference green species). (B) A representative simulated data, where residual synergy in molecular interaction appears (n = 2 of 20). (C) A representative view of molecular distributions from one of nineteen Monte-Carlo simulations where red molecules were selected randomly. (D) The conditional bivariate PCF of the simulated data in panel B. (E) The standardized conditional PCF. (F) The resultant molecular interaction scheme. Residual synergy in molecular interactions was identified for this case by the $g_{GR|Pr(R,B)}$ and $\hat{g}_{GR|Pr(R,B)}$ statistics in panels D and E. (G-L) Case 5 with no synergy where red molecules are distributed randomly with high density: Simulated data and synergy analyses of a case depicted in panel G. Panels G-L are as outlined in Supplementary Fig. 4. (G) The model of molecular interactions, where blue molecules were recruited to red molecules, but the placement of red molecules followed a Poisson distribution (and was thus also unrelated to the placement of the reference green species). (H) A representative simulated data, where residual synergy in molecular interaction appears (n = 20 of 20). (I) A representative view of molecular distributions from one of nineteen Monte-Carlo simulations where red molecules were selected randomly. (J) The conditional bivariate PCF of the simulated data in panel B. (K) The standardized conditional PCF. (L) The resultant molecular interaction scheme. Apparent synergy in molecular interactions was identified for this case by the $g_{GR|Pr(R,B)}$ and $\hat{g}_{GR|Pr(R,B)}$ statistics in panels J and K.

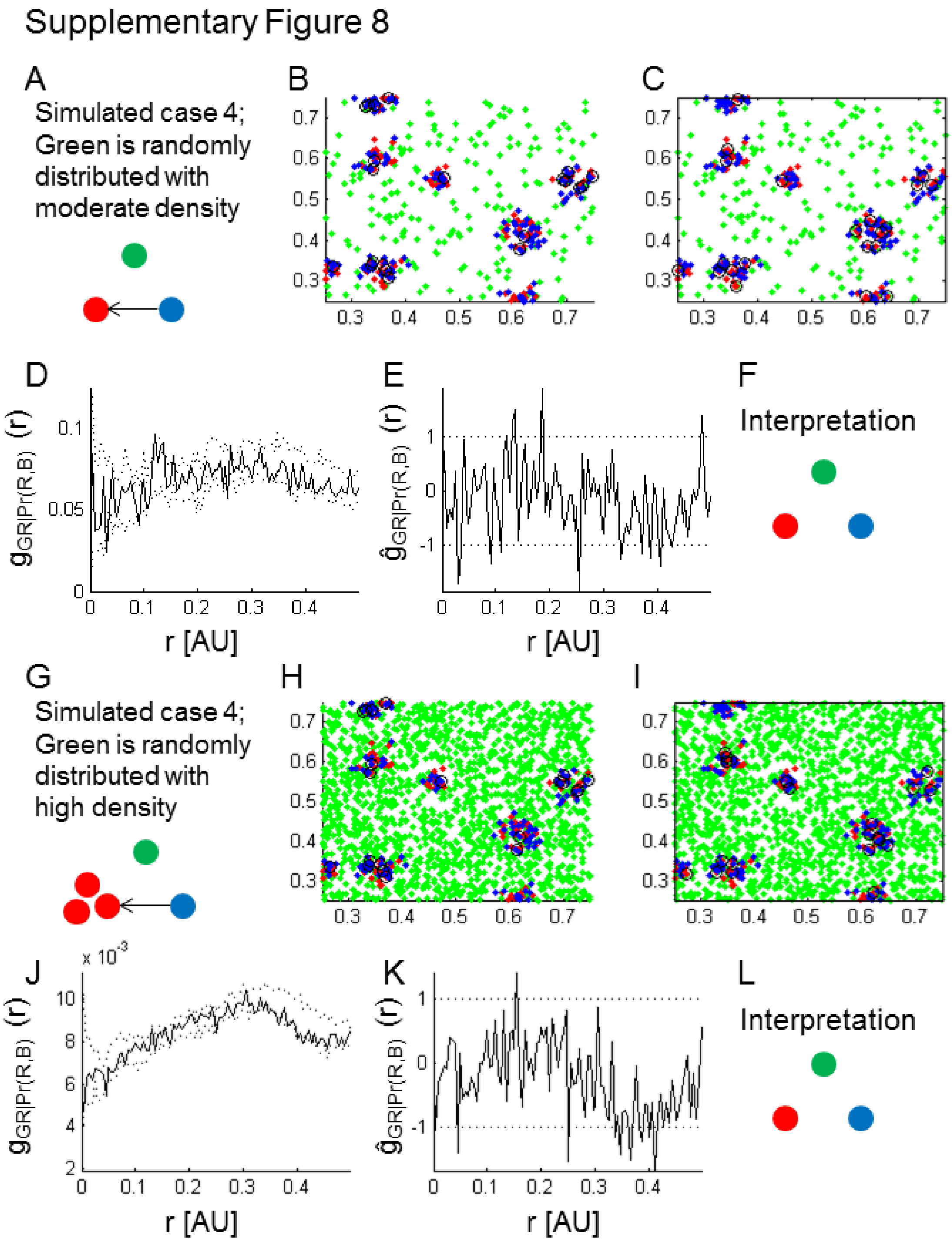
Supplementary Figure 8
A
Simulated case 4; Green is randomly distributed with moderate density
B
C
D
E
F
Interpretation
r [AU]
G
Simulated case 4; Green is randomly distributed with high density
H
I
J
K
L
Interpretation
r [AU]

Supplementary Fig. 8. The interaction synergy analysis relevant to the experimental data is not affected by the random distribution of the reference molecules (cases 4, 6)

(A-F) Case 4 with no synergy where the reference (green) molecules are distributed randomly with moderate density: Simulated data and synergy analyses of a case depicted in panel A. Panels A-F are as outlined in Supplementary Fig. 4. (A) The model of molecular interactions, where blue molecules were recruited to red molecules, but the placement of red molecules was unrelated to the placement of the reference, randomly distributed green species. (B) A representative simulated data (n = 20). (C) A representative view of molecular distributions from one of nineteen Monte-Carlo simulations where red molecules were selected randomly. (D) The conditional bivariate PCF of the simulated data in panel B. (E) The standardized conditional PCF. (F) The resultant molecular interaction scheme. No interactions were identified for this case by the $g_{GR|Pr(R,B)}$ and $\hat{g}_{GR|Pr(R,B)}$ statistics in panels D and E.

(G-L) Case 4 with no synergy where the reference (green) molecules are distributed randomly with moderate density: Simulated data and synergy analyses of a case depicted in panel G. Panels G-L are as outlined in Supplementary Fig. 4. (G) The model of molecular interactions, where blue molecules were recruited to red molecules, but the placement of red molecules was unrelated to the placement of the reference, randomly distributed green species. (H) A representative simulated data, where residual synergy in molecular interaction appears (n = 20 of 20). (I) A representative view of molecular distributions from one of nineteen Monte-Carlo simulations where red molecules were selected randomly. (J) The conditional bivariate PCF of the simulated data in panel B. (K) The standardized conditional PCF. (L) The resultant molecular interaction scheme. No interactions were identified for this case by the $g_{GR|Pr(R,B)}$ and $\hat{g}_{GR|Pr(R,B)}$ statistics in panels J and K.

Supplementary Figure 9

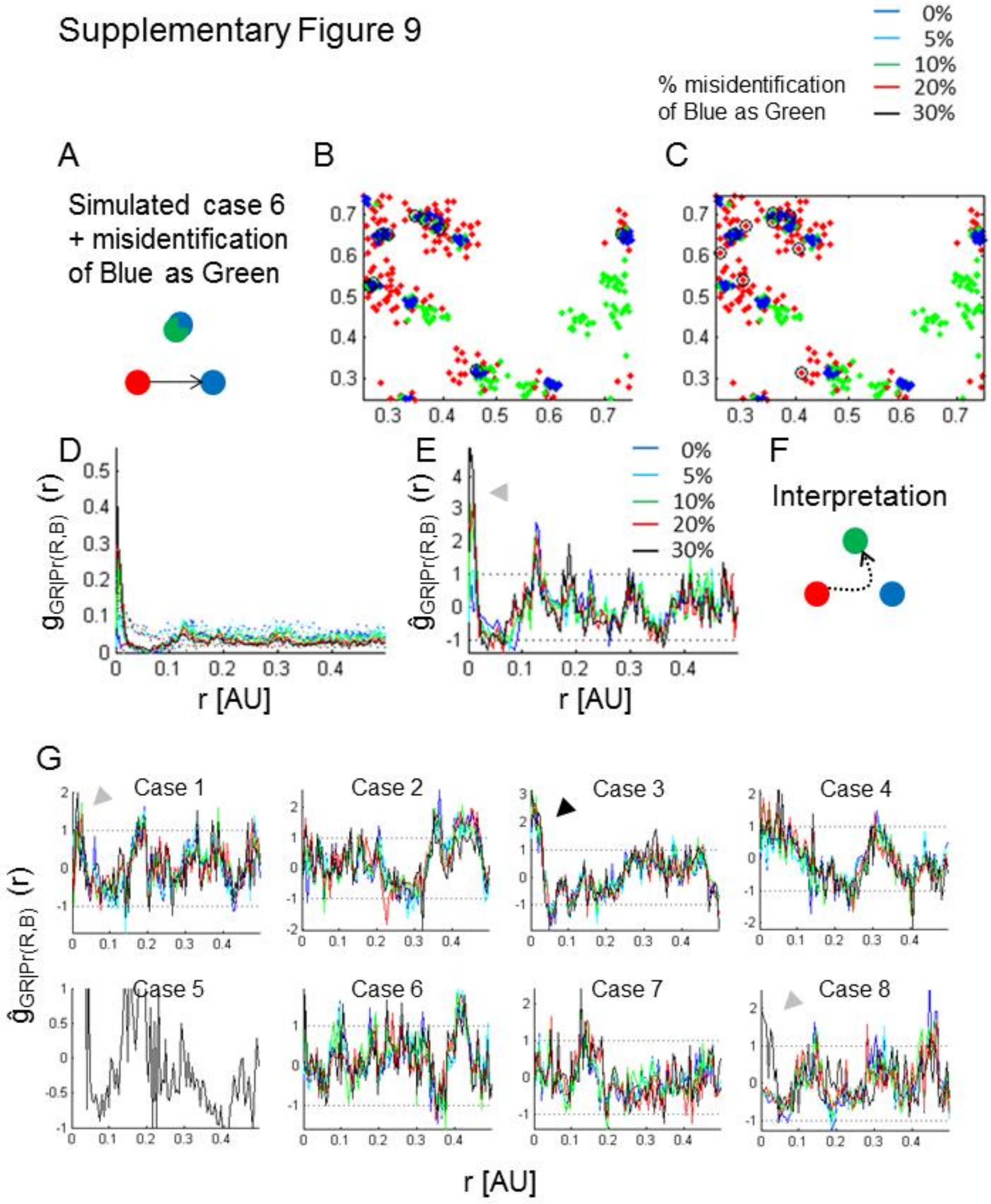

Supplementary Fig. 9. The effect of crosstalk between detection channels on the interaction synergy analysis of all cases

(A-F) Simulated data and interaction synergy analyses of hierarchical molecular clustering following the molecular interaction scheme of case 6 with no synergy as in Supplementary Fig. 6A-F, where blue molecules were misidentified as green molecules. The number of blue molecules used in Supplementary Fig. 4A-F were defined as 100% and 0-30% fractions of these molecules were relabeled as green molecules. The data in these panels is arranged as in panels A-E above and is described briefly below. (A) The model of molecular interactions, where red molecules were recruited to blue molecules, while the placement of blue molecules was unrelated to the placement of the reference green species (from Supplementary Fig. 6 A-F) . Here, the overlapping green and blue circles represent the misidentification of blue molecules as green. (B) A representative simulated data (n = 10) with 30% crosstalk, as described in the SI. (C) A representative realization of molecular distributions from one of nineteen Monte-Carlo simulations where red molecules were selected randomly. (D) The conditional bivariate PCF of the simulated data for a range of cross-talks. (E) Standardized conditional PCFs are shown for a range of cross-talks. Synergies that appear only due to mislabelled molecules are highlighted with a gray arrow-head. (F) The resultant molecular interaction scheme based on the interpretation of the conditional PCFs. Residual synergic recruitment of red molecules to green molecules through blue molecules is marked with a dotted curved arrow between the interacting species.

(G) Interaction synergy analyses of hierarchical molecular clustering following the molecular interaction scheme of all interaction cases (see Supplementary Table 2, and Fig. 5, Supplementary Figs. 4-6) where green molecules were misidentified as blue molecules.

The number of blue molecules used was defined as 100% and 0-30% fractions of these molecules were relabelled as green molecules. Standardized conditional PCFs are shown for a range of cross-talks. Curves are color-coded for the fraction of mislabelled green molecules. Significant synergies are highlighted with black arrow-heads, while synergies that appear only due to mislabelled molecules are highlighted with gray arrow-heads.

Supplementary Figure 10

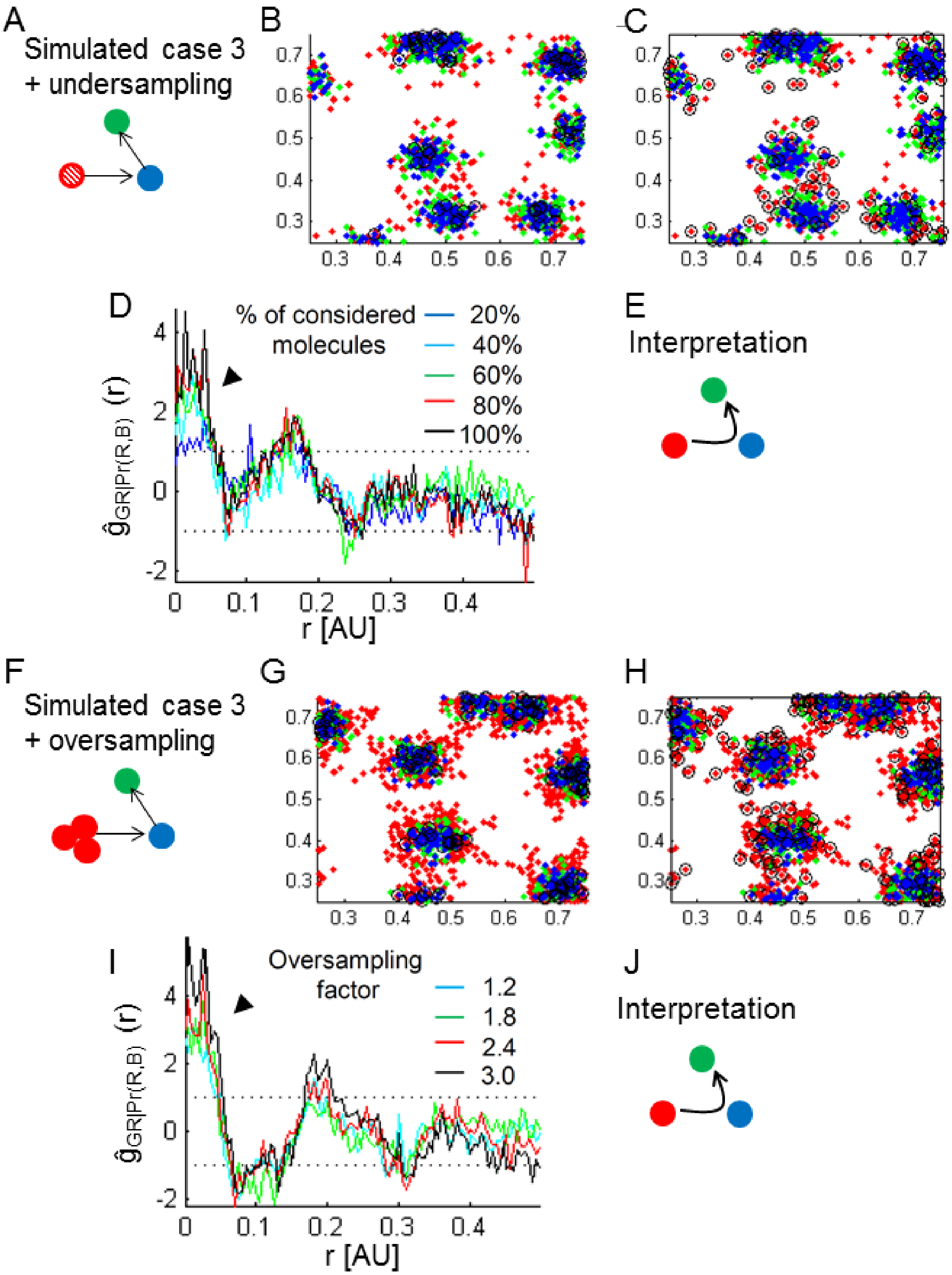

Supplementary Fig. 10. The interaction synergy analysis is robust to under- and over-sampling of detected molecules

(A-E) Simulated data and interaction synergy analyses of hierarchical molecular clustering following the molecular interaction scheme of positive interaction synergy shown in Fig. 5A (case 3), when red molecules were randomly under-sampled. (A) The model of molecular interactions where blue molecules (blue filled circle) were recruited directly to the reference green molecules (green filled circle). Red molecules (green filled circle) were then recruited to blue molecules. Here, dashed filling of red circles represents under-sampling of the red molecules (as discussed section on simulations of the SI). (B) A representative simulated data (n = 10) with consideration of only a 20% subset of the red molecules, which was randomly selected the whole set of red molecules. (C) A representative view of molecular distributions from one of nineteen Monte-Carlo simulations where red molecules were selected randomly. (D) The standardized conditional bivariate PCFs of the simulated data in panel B (as defined in Eqs. 7-10 in the SI), where the dotted black lines are the 95% confidence intervals due to the Monte-Carlo sets (as shown in panel C). Curves are shown for a range of considered fractions of red molecules. Significant interaction synergy is highlighted with a black arrow-head. Even when only 20% of the red molecules were considered, a small degree of interaction synergy could still be observed. (E) The resultant molecular interaction scheme based on the interpretation of the conditional PCFs in panel D. Synergic recruitment of red molecules to green molecules through blue molecules is marked with a curved arrow between the interacting species.

(F-J) Simulated data and interaction synergy analyses of hierarchical molecular clustering following the molecular interaction scheme of positive interaction synergy as in Fig. 5A, Supplementary Fig. 9A, where red molecules were over-counted or self-aggregated. The

number of red molecules used in Supplementary Fig. 9A-F were defined as 100% and more red molecules were added to give oversampling of 120%-300%. The data in these panels is arranged as in panels A-E above and is described briefly below. (F) The model of molecular interactions where red and blue molecules (red and blue filled circle) were recruited to an undetected species (open circle), and where green molecules (green filled circles) were recruited through blue molecules to the undetected species. Here, the multiple red circles represent over-sampling or aggregation of the red molecules. (G) A representative simulated data (n = 10) with 300% oversampling of the red molecules, as described in the SI. (H) A representative realization of molecular distributions from one of nineteen Monte-Carlo simulations where red molecules were selected randomly. (I) Standardized conditional PCFs are shown for a range of over-counted red molecules. Significant interaction synergy is highlighted with a black arrow-head. (J) The resultant molecular interaction scheme based on the interpretation of the conditional PCFs. Synergic recruitment of red molecules to green molecules through blue molecules is marked with a curved arrow between the interacting species despite the high level of oversampling.

Supplementary Figure 11

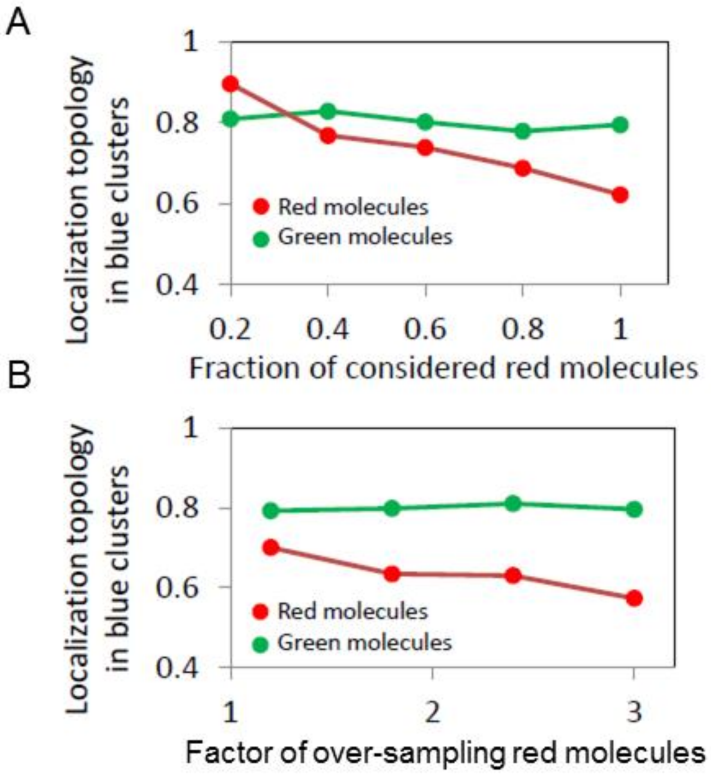

Supplementary Fig. 11. The Topology analysis is robust to under- and over-sampling of detected molecules

(A) The localization topology as a function of the fraction of red molecules after their random under-sampling (see part on interaction synergy simulations in the Materials and Methods section of the SI for further details). (B) The localization topology as a function of the fraction of red molecules after their over-sampling in an aggregated state (see part on interaction synergy simulations in the SI, and main text for further details).

Supplementary Figure 12

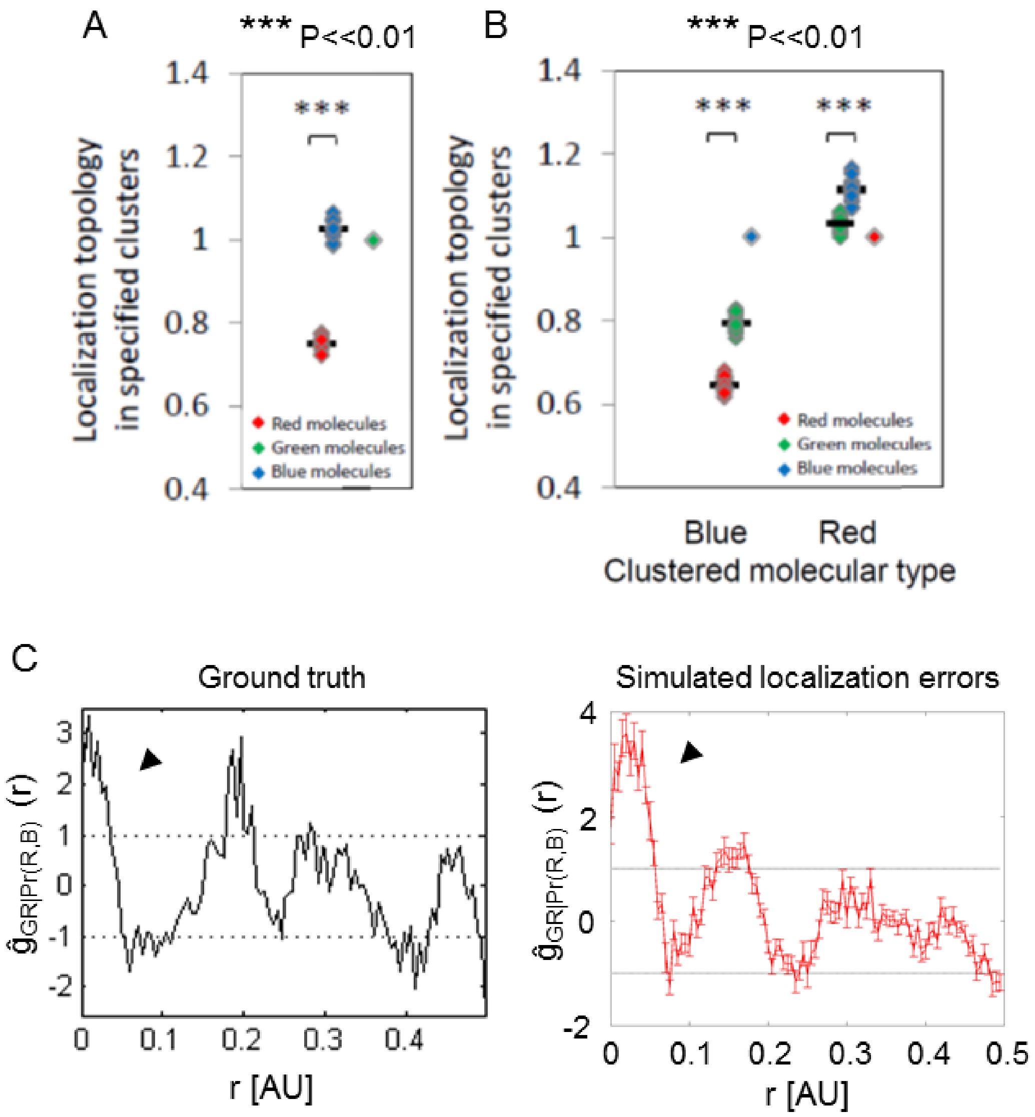

Supplementary Fig. 12. The Topology and interaction synergy analyses are robust to localization errors of detected molecules and to the choice of reference species

(A) The results of 20 simulated realizations where we introduced random errors (drawn from a Gaussian distribution with 1σ matching the experimental errors) to the localizations of all molecules. Simulated molecular patterns were according to case 3 described in Fig. 5A-F. The localization topology was calculated separately for the molecular species in each of the 20 simulations, while molecular species of reference (Green) was assigned a value of 1. *** indicates $p << 0.01$. The p-value was calculated using a two-tailed T-test for unequal variances for the Red and Blue molecules. (Note that SEM error-bars were smaller than a single experimental point in all cases and are not shown). (B) The calculation of localization topology was repeated while selecting each time a different molecular species (namely, either Red or Blue) as the putative center of the cluster. The selected molecular species was given a localization topology measure of 1 and the localization topology was calculated for the two remaining species. In each case, Topology measure was calculated for 20 simulated realizations where we introduced random errors, as in Panel A. P-values were calculated as for the data in panel A. Thus, the shown data accounts for the effects of both localization errors and the choice of reference species on the topology analyses. (C) The average of the 20 standardized conditional PCFs is shown for the 20 simulated realizations (as in panels A and B) that included the localization errors (right panel). The standardized conditional PCFs of the ground truth data with no localization errors (appearing in Fig. 5E) is shown in the left panel for comparison. Error bars indicate 2 SEMs. Significant interaction synergy is highlighted with a black arrow-head.

Supplementary Table 1

Step	Duration [s]	Task	Comments
1	60-120	Drop cells	Cell spread in bright field
2	10	Photoactivate Dronpa	Weak 405nm
3	10	Image Dronpa	Exc. 488nm
4	3	Autofocus	
5	10	Photoactivate PA-GFP and PAmCherry	Dronpa photo-bleached
6	3	Autofocus	
7	10	Image PAmCherry	Exc. 561nm
8	3	Autofocus	
9	10	Image PAGFP	Exc. 488nm
	~5min	Go to Step 5	

Supplementary Table 1. MC-PALM acquisition sequence

A description of the steps of MC-PALM imaging of cells expressing proteins conjugated to the PAFPs Dronpa, PAmCherry and PAGFP. The durations described here apply to live cell imaging, however in fixed cells there were no restrictions on the imaging times.

Case	R	G	B	Expected synergy.	Resulted synergy.	Figure	Comments
1	I(G)	O	I(G)	-	-	S4A-F	
2	I(G)	O	I(R)	-	-	S4G-L	[2]
3	I(B)	O	I(G)	V	V	3A-F, S10	
4	-	O	I(R)	-	-	S5A-F,S7	Flat cond. PCF, [3]
5	-	O	I(G)	-	-	S5G-L,S8	Flat cond. PCF, [1]
6	I(B)	O	-	-	-	S6A-F, S9A-F	Flat cond. PCF, [1]
7	I(G)	O	-	-	-	4G-L	[3]
8	-	O	-	-	-	S6G-L	Flat cond. PCF

Legend:
"R", "G", "B" – the three molecular species, colored in Red, Green or Blue.
"O" – the reference species ("G")
"I(X)" – recruitment to species X (either "R", "G" or "B").
"-" in columns "R", "G" or "B"– unrelated placement of the species to the reference species ("G")
"-" in "expected"/"resulted synergy" columns – no interaction synergy is expected/detected.
"v" in "expected"/"resulted synergy" columns – interaction synergy is expected/detected.

Comments:
[1] This case might show residual interaction synergy if the Blue molecules are more tightly recruited to Green molecules than the recruitment of Red molecules to Green molecules (i.e. $g_{RB} > g_{RG}$). A Flat conditional bivariate PCF indicates false detection of synergy.

[2] We study here only the synergy in the recruitment of Red molecules via Blue molecules to Green molecules, thus do not expect to detect interaction synergy in this case.

[3] May show 'apparent' interaction synergy for randomly placed molecules of species Red or Blue. A Flat conditional bivariate PCF indicates false detection of synergy.

Supplementary Table 2. Summary of synergy in simulated cases of trivariate molecular interactions

This table summarizes the synergy expectation and results for all cases of trivariate molecular interactions, considering a specific reference species. The interaction synergy is calculated between green and blue molecules in the recruitment of red molecules (through the conditional PCF $g_{G,R|Pr(R,B)}$); that is whether red molecules that are close to blue ones show increased interaction with green molecules as well. The individual presentations of these results in figures along the manuscript are specified. See section on Interaction synergy simulations in the SI for discussion on the interpretation of cases that show flat conditional bivariate PCFs.

Supplementary Methods

Cloning. Proteins tagged with the photoactivatable fluorescent proteins (PAFPs) Dronpa (MBL International Corporation), PAmCherry and PAGFP were generated in EGFP-N1 or EGFP-C1 vectors (Clontech). PAFPs genes were cut from a vector through digestion with restriction enzymes (typically AgeI and NotI, XbaI or BsrGI). PAFP genes then served to replace existing fluorescent proteins (FP) in previously used constructs (*1, 2*) using similar digestion reactions and ligation of the PAFP insert (Quick-ligation kit, New England BioLabs). Specifically, newly generated constructs for this study included PLCγ1-Dronpa, PLCγ1-PAGFP, PAGFP-Actin, PAmCherry-Actin, Dronpa-Actin, VAV1-PAmCherry, TCRζ-PAGFP, and SLP-76-PAGFP. Validation of cloning was done by restriction digestion analyses and DNA sequencing of the inserts. Sequencing was performed by the NCI DNA sequencing core facility.

Jurkat T cell cultures and transfections. E6.1 Jurkat T cells were transfected with DNA using a nucleofector shuttle system, program H-10 and the Amaxa T-kit (Lonza). Transiently transfected cells were maintained in transfection medium, sorted for positive expression of PAmCherry, Dronpa or PAGFP chimeras and imaged within 48-72 hours from transfection. Stable cell lines were created by selection with Geneticin at 1.5mg ml^{-1} (G418, Invitrogen). After 2-3 weeks, the cells were sorted and single clones were grown in 96 well plates. After 3 additional weeks, the extent of protein expression was checked by flow cytometry (see details below). Cells were then evaluated using biochemistry assays, flow cytometry, confocal microscopy (510 LSCM, Zeiss) and epifluorescence, TIRF and PALM imaging, as described below.

Flow cytometric analyses and sorting. Cells were sorted by flow cytometry to obtain positive cells from transiently transfected cells and to create stable cell lines with

uniform expression. Sorting was performed by the NCI flow cytometry core facility using a Moflo Astrios high speed cell sorter (Beckman Coulter) and Summit software. Cells expressing Dronpa-tagged proteins were sorted for their GFP-like green emission (488 nm excitation, 500-520 nm emission). The emission of PAGFP was typically faint and required extended photoactivation, as described below. When sorting samples that expressed both Dronpa- and PAGFP-tagged proteins for MC-PALM, we relied on stable expression of PAGFP-tagged proteins and transient expression of Dronpa-tagged proteins. PAmCherry transfected cells were first activated using a light emitting diode (LED) source (a blue light source, CoolLED, PE-100, HITECH Instruments for 10 min). The emission of the LED light source was directed onto a single well of a 24-well plate that contained the cells, suspended in a buffer without phenol red (*1*). Cells were then sorted for red fluorescence (561 nm excitation, 560-600 nm emission). Results were further analyzed with FlowJo (TreeStar).

Sample preparation. The preparation of coverslips for imaging spread cells followed a previously described technique (*3*). Briefly, for diffraction limited and PALM imaging, coverslips (#1.5 glass chambers, LabTek) were washed with acidic ethanol at room temperature (RT) for 10 min; liquid was then aspirated and coverslips were dried at 40°C for 1 hour. These coverslips were then coated with 100 nm gold beads (Microspheres-nanospheres) that had been sonicated and diluted x10 in methanol. Cleaned coverslips with beads were incubated at RT for 15 min with 0.01% poly-L-lysine (Sigma) diluted in water. Liquid was aspirated and coverslips were dried at 40°C for 12 hours. Coverslips were subsequently incubated with stimulatory or non-stimulatory antibodies at a concentration of 10 $\mu g\ ml^{-1}$ (unless specified otherwise) overnight at 4°C or 2 hours at 37°C. Finally, coverslips were washed with PBS. Throughout the study we used the following stimulatory antibodies: purified mouse αhuman αCD3 (clone Ucht1) and αCD45 (BD Biosciences). A

few hours before imaging, cells were resuspended in imaging buffer at a concentration of 1 million per 150 µl and 100,000-500,000 cells were dropped onto coverslips for PALM or diffraction limited imaging, incubated at 37°C for the specific spreading time (typically 3 min) and fixed with 2.4% PFA for 30 min at 37°C.

Confocal imaging - Confocal imaging was performed using a 510 LSCM confocal microscope using a 63X, 1.4 NA objective (Zeiss).

MC-PALM imaging - Multi-Color Photoactivated localization microscopy (MC-PALM) imaging was conducted similarly to the imaging previously described (*1*), using a total internal reflection (TIRF) Nikon microscope. However, here the imaging sequence of tagged proteins followed the sequence described in Supplementary Table 1. As a first step, Dronpa-tagged proteins were imaged using continuous and low intensity ~340 nm illumination of an arc lamp (DAPI cube) and laser excitation at 488 nm in TIRF mode. Dronpa-tagged molecules were imaged for 10 sec for live-cell imaging, or the depletion of their emission, as identified by the loss of fluorescence, for fixed-cell imaging. For cells expressing higher levels of Dronpa-conjugated proteins, it is critical to remove as many Dronpa molecules as possible to avoid bleedthrough in the PAGFP images. The focus of the microscope was then adjusted using the PerfectFocus system of the microscope. Similar focus adjustments were also performed after each imaging step that followed, as described below. After imaging Dronpa, the sample was illuminated with maximal intensity of the Arc lamp illumination at ~440 nm (CFP cube) for 10 sec for imaging living cells and 10-20 sec for imaging fixed cells. This step served to activate PAmcherry- and PAGFP-tagged proteins and to photobleach residual Dronpa-tagged proteins. Longer activation times are more efficient at photobleaching Dronpa. We next imaged PAmCherry-tagged proteins, following by imaging PAGFP-tagged proteins. Each imaging step took 10 sec for live-cell

imaging and ~30 sec for fixed cells. The sequence of steps of photoactivation, imaging PAmCherry and PAGFP were then repeated multiple times: 6 times in total for live-cell imaging or until depletion of emission from all molecules for fixed-cell imaging.

As with other imaging technique, our MC-PALM approach requires the complexes under study to be relatively stable through the effective frame time of imaging (~20-30 sec). Shortening the acquisition time could be achieved by using faster cameras, brighter fluorophores, enhancing excitation of fluorophores using brighter lasers, and using algorithms that can detect molecules with overlapping point-spread functions (e.g. (*4*)). Also, it should be noted that Dronpa photobleaching is essentially accomplished already at the beginning of the imaging sequence and that its fluorescence decays fast and exponentially upon photobleaching (with a lifetime $\tau < 2$sec). Thus, Dronpa photobleaching could be shortened to further accelerate imaging, esp. after the first cycle of imaging.

Bleed through is an important consideration in all imaging experiments. It affects not only our attempts to image two different green photoactivatable proteins, but occurs in imaging of red and green photoactivatable proteins and multi-color STORM experiments as well. To insure that Dronpa is not residing in a dark state that can be reactivated, the sample should be treated with a brief pulse of activating light that will only activate Dronpa molecules. If green fluorescence is seen, the sample should be photobleached for additional time and checked again. Fluorescence from Dronpa molecules also decays more quickly than fluorescence from PA-GFP so a delay can be introduced between the start of visualization of green fluorescence and recording of the images to further reduce cross-talk between Dronpa and PA-GFP. A control series should be recorded with a brief excitation that will maximally activate Dronpa molecules without activating PA-GFP to demonstrate that in each sample there is no remaining Dronpa fluorescence.

Throughout imaging, 100nm gold beads (Microspheres-Nanospheres) were used as fiduciary markers to account for drift and for registration of the MC-PALM channels. Typical registration between the MC-PALM channels was < 10 nm across the imaging field. Sample sizes were chosen to account for cell to cell variability, within experimental constraints. Notably, each measurement of individual cells contained tens to hundreds of molecular clusters, over which the localization topology and interaction synergy statistics were calculated (as detailed below).

Analyses

PALM rendering - movies generated by MC-PALM imaging were analyzed by the PeakSelector software 5 for the identification of individual peaks in the movie frames. Next, peaks were grouped and assigned to individual molecules for rendering of the MC-PALM images. Peak grouping used a distance threshold and a temporal gap to account for possible molecular blinking (*5*). A range of temporal gaps were considered for each fluorophore separately in order to minimize possible overcounting of molecules. Individual molecules are presented in MC-PALM images with intensities that correspond to the probability density values of their fitted Gaussian with respect to the maximal probability density values detected in the field.

Localization topology - To determine the localization topology of molecules around LAT clusters we first generated 3 binary images [$R(i,j),G(i,j),B(i,j)$] matching the 3 channels imaged by MC-PALM. In each binary image, a disk with a diameter equivalent to 20 nm and intensity equal to 1 marked the positions of individual molecules, as identified by the PeakSelector software (*5*). Here, i and j are the pixel indexes of the image. We discuss considerations for choosing the diameter of the disks for the different binary images at the end of this sub-section.

We next performed a Euclidean distance transform (*DT*) (*6*) of the negative of the binary image that was created from the rendered image of LAT molecules. We further normalized the resultant image (Eq.1).

$$(1)\qquad G^{DT}(i,j) = \frac{DT[G(i,j)]}{\max(DT[G(i,j)])}$$

The normalized distance-transformed image, G^{DT} , provided a watershed description (*7*) of LAT clusters, with values ranging between 0 and 1. Here, the highest values mark the points furthest from the outside boundary of LAT clusters and lower values mark points that are closer to the boundary. This image was used to produce weighted images by pixel-wise multiplication of the individual binary images of the 3 different channels by the normalized distance-transformed image, G^{DT}. The localization topology (*LT*) values were calculated as the average intensity throughout the weighted binary images divided by the average intensity of the weighted binary image of the green LAT channel (Eq.2).

$$(2)\qquad \begin{aligned} LT_R &= \langle R(i,j)G^{DT}(i,j)\rangle / \langle G(i,j)G^{DT}(i,j)\rangle \\ LT_B &= \langle B(i,j)G^{DT}(i,j)\rangle / \langle G(i,j)G^{DT}(i,j)\rangle \\ LT_G &\equiv 1 \end{aligned}$$

All image transforms and analyses were calculated using custom codes written in Matlab (MathWorks) and its Image analysis toolbox. Figures were created using Matlab (for images) or Prism (for topology results related to multiple cells).

The diameters of the disks that are used for determining the topology measure require the following considerations. First, we chose the disk size for representing the reference molecular species (i.e., the species that clusters). This disk size depends on the local concentration of molecules that serve to define the cluster of reference. From simple geometrical considerations, we can define the inter-particle distance, $di = (Ai / Ni)^{0.5}$, where

Ni is the number of molecules in cluster *i* and *Ai* is the area of the cluster. A more general estimate of *d* over multiple clusters can be achieved by counting the number of molecules over a wider study region (that contains multiple clusters) of area *A*. Then $d = (A / N)^{0.5} / g_{11}(0)^{0.5}$, where $g_{11}(0)$ is the value of the univariate pair-correlation function at a distance scale of 0 and accounts for the higher density of molecules within clusters. Note that we assume here no over-sampling of molecules, as we solve this problem using a grouping approach of our PALM data. Corrections for over-sampling through related imaging by dSTORM can be made following (*8*, *9*). Next, we would like to assign a disk of radius *r* to each molecule, thus setting a distance Δ between two adjacent disks of $\Delta = di - 2r$. For our morphology analysis we would like to get $\Delta \sim 0$. Thus, *r* is chosen such that $r = 0.5(A / N)^{0.5} / g_{11}(0)^{0.5}$, when taking measurements across a wide region of interest (and for multiple clusters within). Typical values in our experiments yield 3,000-10,000 molecules across a study section of 40 μm^2 and $g_{11}(0) \sim 2 - 6$. These provide typical values for *r* of 15-30 nm for our PALM images of LAT. We chose 20 nm for our analyses in the study. We would like to stress that our segmentation technique here is essentially a nearest-neighbour clustering approach and that other segmentation procedures for clusters are directly applicable at this stage of the analysis (e.g. by intensity or density thresholding).

Second, the radii of the disks that mark the two other molecular species should be significantly smaller than the cluster size to avoid averaging out of their localization (and scores) with respect to the cluster. As a lower limit, the disks can be limited by the localization error. Note that this is a soft limit, as these errors are uncorrelated and isotropic, so they average out over many molecules and many clusters. In our assays, a diameter of 20 nm served nicely also for the disks describing the two molecular types, although smaller disks can also be used. Choosing different disk sizes at this stage under the specified considerations would moderately change the absolute values of the topology

measure but will not affect the relative localizations (or hierarchy) of the 2 species in respect to the clusters (see a related example in Supplementary Fig. 11, panels A,B).

Interaction synergy - We quantified evidence for the synergy of molecular interactions in their binding to a third molecular species, based on their positions in the MC-PALM image of individual cells. This analysis was applied to study regions that covered most of the apparent footprint of the cells. We will describe the algorithm by referring to an example where we study the interactions between type 2 and type 3 molecules, upon their binding to type 3 molecules. The set of x,y coordinates of each molecular species is denoted here by S_i, where i = 1,2,3. First, we identified the proximity of molecules from two species of interest (e.g., Type 2 and Type 3) using the Boolean function Pr (Eq.3). Pr is calculated for each pair of molecules (s_2, s_3) from S_2 and S_3, as follows:

$$(3) \qquad \Pr(s_2, s_3) = \begin{cases} 1 & Dist(s_2, s_3) \le d_{th} \\ 0 & Dist(s_2, s_3) > d_{th} \end{cases}$$

where the operator $Dist(s_i,s_j)$ is the Euclidean distance between the points *si* and *sj* and where d_{th} is the threshold for defining proximity. The value of this threshold, d_{th}, was varied between 20 nm and 200 nm to check the robustness of our analyses approach (Supplementary Fig. 1). A threshold of 40 nm was then chosen to select the interacting Type 2 molecules, using the function Pr to obtain a subset S_2' of the points of type 2 (namely S_2), following the set-builder notation of Eq.4. Notably, in our PALM measurements the localization uncertainty of individual molecules peaked at ~25nm for all different colors (see Fig. 1D). Thus, the 40 nm threshold was about twice the size of the uncertainty of molecular localization in our study. Another consideration for setting the

proximity threshold involves the molecular density in the data, as discussed above for the localization topology.

(4) $$S_2' = \{ s_2 | \exists s_3 \in S_3 \quad such \quad that \quad \Pr(s_2, s_3) = 1 \}$$

Together, eqs.3 and 4 state that s_2 is included in S'_2 if there exists at least one proximal molecule s_3 from S_3 that lies below the threshold distance d_{th} from s_2.

Next, we calculated the *conditional* bi-variate pair-correlation function (PCF; $g_{12|\Pr(2,3)}(r)$) of the selected subset of Type 2 molecules, S_2', with a third molecular species of Type 1. Following a similar notation to Wiegand (*10*), a bivariate PCF can be calculated for a pixelated image using the following definitions:

(5) $$g_{12}(r) = \frac{A}{n_2} \frac{\frac{1}{n_1}\sum_{k=1}^{n_1} Pnts\left[S_2, R_{1,k}^{w}(r)\right]}{\frac{1}{n_1}\sum_{k=1}^{n_1} Area\left[R_{1,k}^{w}(r)\right]}$$

where, $R_{1,k}^{w}(r)$ is the ring with radius *r* and width *w* centered on the *k*'th point of type *2* (here points of type *2* are simply type *i* molecules, or S_2, as defined above). n_i is the total number of points of type *i* in the study region of area *A*. The operator *Pnts* [S_j,*X*] counts the points of type *j*, namely S_j, in region *X*. The operator *Area* counts the number of cells in the region *X*. Similarly, the *conditional* bi-variate pair-correlation function (PCF; $g_{12|\Pr(2,3)}(r)$) is defined in Eq.6. This equation now refers to S_2', the proximity-selected sub-population of S_2, with a total number of molecules of $n_{2'}$.

(6) $$g_{12|\Pr(2,3)}(r) = \frac{A}{n_{2'}} \frac{\frac{1}{n_1}\sum_{k=1}^{n_1} Pnts\left[S_2', R_{1,k}^{w}(r)\right]}{\frac{1}{n_1}\sum_{k=1}^{n_1} Area\left[R_{1,k}^{w}(r)\right]}$$

To check the significance of the interaction synergy, the conditional bivariate PCF was compared to the bivariate PCF of Type 1 and the same number of chosen Type 2 molecules that were randomly spread across the positions of all of the identified type 2 molecules, regardless of their proximity to Type 1 molecules (yielding $\tilde{g}_{12}(r)$ in Eqs.7 and 8 below). A Monte-Carlo simulation was used in this later stage to generate nineteen control sets through the described random placement of molecules and to mark a range of 95% confidence interval, within which the interaction is not significantly synergic.

We also studied the effect of molecular exclusion through our interaction synergy analysis. Specifically, we calculated the conditional bivariate PCF for the non-selected population of molecules (i.e., molecules that have been excluded from the proximity selection; bold black line in Supplementary Fig. 1, panel C). As expected, the conditional bivariate PCF of this population lies beneath the dotted gray lines that represent the 95% confidence interval of no synergy in molecular interactions. This indicates that the non-selected population has negative interaction synergy (i.e. the interaction of these molecules with a molecule of interest is diminished when the exclusion criterion is applied). This observation exactly matches our expectation from our interaction synergy statistics.

To further compare the synergy of the molecular interaction within multiple cells, we first standardized the conditional bi-variate PCFs independently for each cell *i* following Eq.7.

$$(7) \qquad \hat{g}_{12|\Pr(2,3)}{}^{i}(r) = \frac{g^{i}_{12|\Pr(2,3)}(r) - \left\langle \tilde{g}^{i}_{12}(r) \right\rangle}{\tilde{\sigma}^{i}(r)}$$

where,

$$(8) \qquad \tilde{\sigma}^{i}(r) = \max\left(\left| \left(\tilde{g}^{i}_{12|\Pr(2,3)}(r) - \left\langle \tilde{g}^{i}_{12}(r) \right\rangle \right) \right| \right)$$

Here, $2\tilde{\sigma}^{i}(r)$ denote the 95% confidence interval of the bivariate PCF of cell *i* due to the nineteen simulated random sets of the null hypothesis. We then took the average of the standardized conditional bivariate PCFs and the standard error of the mean (SEM) over multiple *N* cells using the following equations (Eqs.9 and 10):

(9) $$\left\langle \hat{g}_{12|\Pr(2,3)}(r) \right\rangle = \frac{1}{N}\sum_{i=1}^{N} \hat{g}^{i}_{12|\Pr(2,3)}(r)$$

(10) $$SEM(r) = \sqrt{\frac{1}{N^2}\sum_{i=1}^{N} \hat{g}^{i}_{12|\Pr(2,3)}(r)^2}$$

Finally, to report on the synergy of interactions due to multiple cells, the resultant curves of $\left\langle \hat{g}_{12|\Pr(2,3)}(r) \right\rangle$ and SEMs were plotted (bold lines and error bars) along with the normalized 95% confidence interval (dotted gray lines). The departure of the averaged PCF from the confidence intervals serves to reject the null hypothesis, indicating that the binding of Type 1 and Type 2 is promoted by the interaction of Type 2 molecules with molecules of Type 3. Notably, in our robustness analyses (Supplementary Fig. 1), selecting a threshold that excludes proximal molecules resulted in a negative interaction synergy, where the bold curves lied below the 95% confidence interval.

Note that generally, for any case of tri-variate molecular interactions there are overall 12 conditional PCF curves that can be calculated (along with matching 12 standardized curves). For instance, for g_{12} there are 4 such statistics: $g_{12|\Pr(2,3)}$, $g_{21|\Pr(2,3)}$, $g_{12|\Pr(1,3)}$, $g_{21|\Pr(1,3)}$. Similarly, 4 such statistics apply also for g_{13} and g_{23}. Note that the PCFs g_{12} and g_{21} are not symmetric, while the proximity operator Pr is symmetric.

All calculations of PCFs were conducted using a published software (*10*) or custom codes written in Matlab (MathWorks). All curves were plotted in Excel (Microsoft).

Interaction synergy simulations

All simulations were performed using custom algorithms coded in Matlab. For simulating the synergic interactions we started by randomly choosing multiple points as nucleation sites for molecular clusters. We then distributed molecules of a reference species (Green throughout the text) with Gaussian clustering statistics. That is, distributing molecules was performed by randomly choosing their x-y coordinates across the simulated field. Next, the randomly positioned molecules were eliminated across most of the field but were kept with a Gaussian probability around points that served as nucleation sites. This process was repeated until a target number of molecules were kept and present across the field. We next distributed ‘directly recruited molecules’ (either Red, Blue, or both Red and Blue molecules, depending on the simulated case) around the points of attraction with Gaussian clustering statistics. ‘Indirectly recruited molecules’ were simulated, depending on the simulated scenario, by distributing the molecular species (e.g. Red molecules) around another (e.g. Blue) species that was directly recruited to the reference species (always Green in our simulations). The molecular patterns of interactions are depicted in the interaction schemes in panels A and G of Fig. 5, and of Supplementary Figs. 4-10. The Gaussian width of clustering (where applied) was Red - 0.027, Green - 0.017, and Blue - 0.007 in all relevant simulations. ‘Unrelated molecules’ were clustered around randomly (Poisson) distributed centers across the image with no relation to any of the other molecular species. Various recruitment conditions and scenarios served to simulate either a 'positive' case of interaction synergy (Fig. 5A-F, Supplementary Fig. 10) or ‘negative’ cases (Fig. 5G-L, Supplementary Figs. 5-6) where no interaction synergy was present. Each case was simulated 10-20 times. The results of the simulations are presented in Fig. 5, Supplementary Figs. 4-10 and are summarized in Supplementary Table 2. Our results

indicate that our interaction synergy analysis could distinguish the case where synergy in molecular interactions existed (case 3) from the other cases where interaction synergy did not exist (cases 1,2, 4-9).

We note that negative cases can be generally divided in to two parts. One part of these cases (cases 1,2 and 7 in Supplementary Table 2) involve the direct recruitment of the Red species to the reference Green molecules without the involvement of Blue molecules (Fig. 5G-L, Supplementary Fig. 4). For these cases the conditional PCFs turn upwards at small length-scales, indicating correlated distribution of Red and Green molecules (Fig 5J, Supplementary Fig. 4D, J). This observation assists in the correct interpretation of these cases (Fig. 5L, Supplementary Fig. 4F,L).

In the other cases (cases 4,5,6 and 8 in Supplementary Table 2), Red molecules are unrelated to the reference Green molecules (Supplementary Figs. 5 and 6). In these cases, the conditional PCFs are flat (Supplementary Figs. 5D,J and 6D, J), indicating no molecular interactions between Red and Green molecules. Again, this observation assists in the correct interpretation of these cases (Supplementary Figs.5F,L and 6F,L). In these cases, the recruitment pattern of the Blue molecules cannot be interpreted without the calculation of additional statistics of conditional PCFs, as discussed in the sub-section on interaction synergy. Such statistics can be readily calculated. For instance, we verified, but do not show, the PCFs statistics of the form $g_{GB|Pr(R,B)}$ that captures the synergic recruitment of blue molecules, via red molecules, to green molecules (case 2, Supplementary Fig. 4G-L). However, we limited our simulations and discussion here to the case of synergic (or non-synergic) recruitment of Red molecules, via Blue molecules to reference Green molecules. For these cases, the PCFs of the form $g_{GR|Pr(R,B)}$ are of most interest and the other 11 PCFs are not shown.

To check the robustness of the statistics for all possible configurations of molecular arrangements, we further simulated additional 7 cases where Red molecules served as the reference species and another 7 cases where Blue molecules served as the reference species (in analogy to cases 1-7 in Supplementary Table 2; case 8 is trivial and its analogous cases were not simulated). Our simulations indicated similar robustness of our statistics in distinguishing cases of positive interaction synergy from negative ones (data not shown).

Limitations to the interaction synergy analysis

It should be noted that on occasion (<10% of the simulations for the indicated cases in Supplementary Table 2), residual interaction synergy was observed also for some of the negative cases. This occurs due to the stochastic nature of our simulations, when Blue molecules were distributed by chance closer to the reference Green species than Red molecules (namely, when $g_{RB} > g_{RG}$).

One such example of special interest is illustrated in Supplementary Fig. 7A-F. In this example we simulated a scenario of case 5 where Blue and Green molecules were co-clustered and Red molecules showed a random distribution that was independent on either Blue or Green molecules. Here, we observed positive values of interaction synergy in the standardized conditional bivariate PCF (Supplementary Fig. 7E), for two events in this example. The presence of these rare events makes the interaction synergy seems very noisy. Increasing the concentration of the randomly distributed Red molecules beyond a threshold density resulted in an 'apparently' robust synergic interaction (Supplementary Fig. 7G-L). The univariate PCF of the randomly distributed Red molecules in both cases is completely flat (*10*). Thus, such cases, where one of the species is randomly distributed, can be easily identified and interpreted by univariate PCFs of each species.

For the trimolecular interactions shown in case 5, a threshold for the density of the randomly distributed species can be estimated, as follows. Consider a region of interest of area A (e.g. 1 μm^2). Assume next a proximity threshold of d (e.g. 40nm). Then, the number of 'boxes' available for the random species is $\mathrm{N_T = A/d^2}$. We first distribute randomly $\mathrm{N_3}$ molecules of species 3 in the $\mathrm{N_T}$ boxes. We then distribute randomly $\mathrm{N_2}$ molecules of species 2. The probability of encounter between a single molecule of species 3 and any of the molecules of species 2 is $P_{\mathrm{encounters}} = \mathrm{N_2/N_T}$. The number of actual encounters between molecules of species 2 and 3 can now be estimated by:

$$\mathrm{N_{encounters}} = P_{\mathrm{encounters}}\mathrm{N_3} = \frac{\mathrm{N_3N_2}}{\mathrm{N_T}} = \frac{\mathrm{N_3N_2d^2}}{\mathrm{A}}$$

From simulations, we find that $\mathrm{N_{encounters}} > 10$ yields persistent 'apparent' cooperativity. In our experiments, typical areas of study regions were ~100 μm^2, and d was chosen as 40nm. Such parameters impose a constraint on the product of $\mathrm{N_3N_2}$ in the study region of 2.5*10^6, which limits the analyses in case 5 to relatively low concentrations. Typical concentrations of molecules in our study range between 20 and 500 molecules per μm^2, depending on the molecular species under study. A similar argument and the determination of molecular abundance threshold apply to case 7.

Importantly, such incidences of 'apparent' interaction synergy where one of the molecular species under study is randomly distributed are essentially *irrelevant to our experimental data*, as explained in the following. In our experimental data, Green molecules serve as reference molecules. When we randomly distribute them in the study region (Supplementary Fig. 8), it can be easily seen that both the non-standardized (Supplementary Fig. 8D,E) and the standardized conditional bivariate PCFs indicate no synergy in the interaction. This result is insensitive to the density of the randomly distributed reference molecule and holds for both moderate (Supplementary Fig. 8A-F) and high densities (Supplementary Fig. 8G-L). We conclude that our synergy analysis is valid when using the most abundant and homogeneously distributed species as a reference species, regardless of its density. Caution should be taken when either one of the other two species is randomly

distributed (as monitored by its univariate PCF) and chosen as the reference species, as described above.

To summarize, we recommend the following restrictions and guidelines for the application of the synergy analysis as follows:

1. Limit the cross-talk to < 5- 10% between all channels.

2. Limit the analysis of a randomly (or largely homogeneous) distributed species to a case where it is chosen as the reference species. Alternatively, calculate the threshold on the product of the abundance of the two species for which proximity is studied, $\mathbf{N_3 N_2}$, where N_1 is the reference species and either one of N_2 or N_3 is the randomly distributed species. The random distribution of any species is easily identified through its flat univariate PCF (*10*).

3. Flat non-standardized bivariate PCF curve indicates no interaction between the species under study.

Supplementary References

1. E. Sherman *et al.*, Functional nanoscale organization of signaling molecules downstream of the T cell antigen receptor. *Immunity* **35**, 705 (Nov 23, 2011).
2. A. Braiman, M. Barda-Saad, C. L. Sommers, L. E. Samelson, Recruitment and activation of PLC gamma 1 in T cells: a new insight into old domains. *Embo Journal* **25**, 774 (Feb 22, 2006).
3. S. C. Bunnell, V. A. Barr, C. L. Fuller, L. E. Samelson, High-Resolution Multicolor Imaging of Dynamic Signaling Complexes in T Cells Stimulated by Planar Substrates. *Sci. STKE* **2003**, pl8 (April 8, 2003, 2003).
4. S. J. Holden, S. Uphoff, A. N. Kapanidis, DAOSTORM: an algorithm for high-density super-resolution microscopy. *Nature Methods* **8**, 279 (Apr, 2011).
5. E. Betzig *et al.*, Imaging intracellular fluorescent proteins at nanometer resolution. *Science* **313**, 1642 (Sep 15, 2006).
6. C. R. Maurer, R. S. Qi, V. Raghavan, A linear time algorithm for computing exact Euclidean distance transforms of binary images in arbitrary dimensions. *Ieee T Pattern Anal* **25**, 265 (Feb, 2003).
7. F. Meyer, Topographic Distance and Watershed Lines. *Signal Process* **38**, 113 (Jul, 1994).
8. P. Sengupta *et al.*, Probing protein heterogeneity in the plasma membrane using PALM and pair correlation analysis. *Nat Methods* **8**, 969 (Nov, 2011).
9. S. L. Veatch *et al.*, Correlation functions quantify super-resolution images and estimate apparent clustering due to over-counting. *PLoS One* **7**, e31457 (2012).
10. T. Wiegand, K. A. Moloney, Rings, circles, and null-models for point pattern analysis in ecology. *Oikos* **104**, 209 (Feb, 2004).
